# Supplementary material for: Patronin/Shot Cortical Foci Assemble the Noncentrosomal Microtubule Array that Specifies the Drosophila Anterior-Posterior Axis
Source: Dev Cell. 2016 Jul 11;38(1):61–72. doi: 10.1016/j.devcel.2016.06.010 (PMC4943857; doi:10.1016/j.devcel.2016.06.010)
Supplement: Document S1. Supplemental Experimental Procedures and Figures S1–S5 [file mmc1.pdf]

Developmental Cell, Volume *38*

Supplemental Information

Patronin/Shot Cortical Foci Assemble the Noncentrosomal Microtubule Array that Specifies the *Drosophila* Anterior-Posterior Axis

Dmitry Nashchekin, Artur Ribeiro Fernandes, and Daniel St Johnston

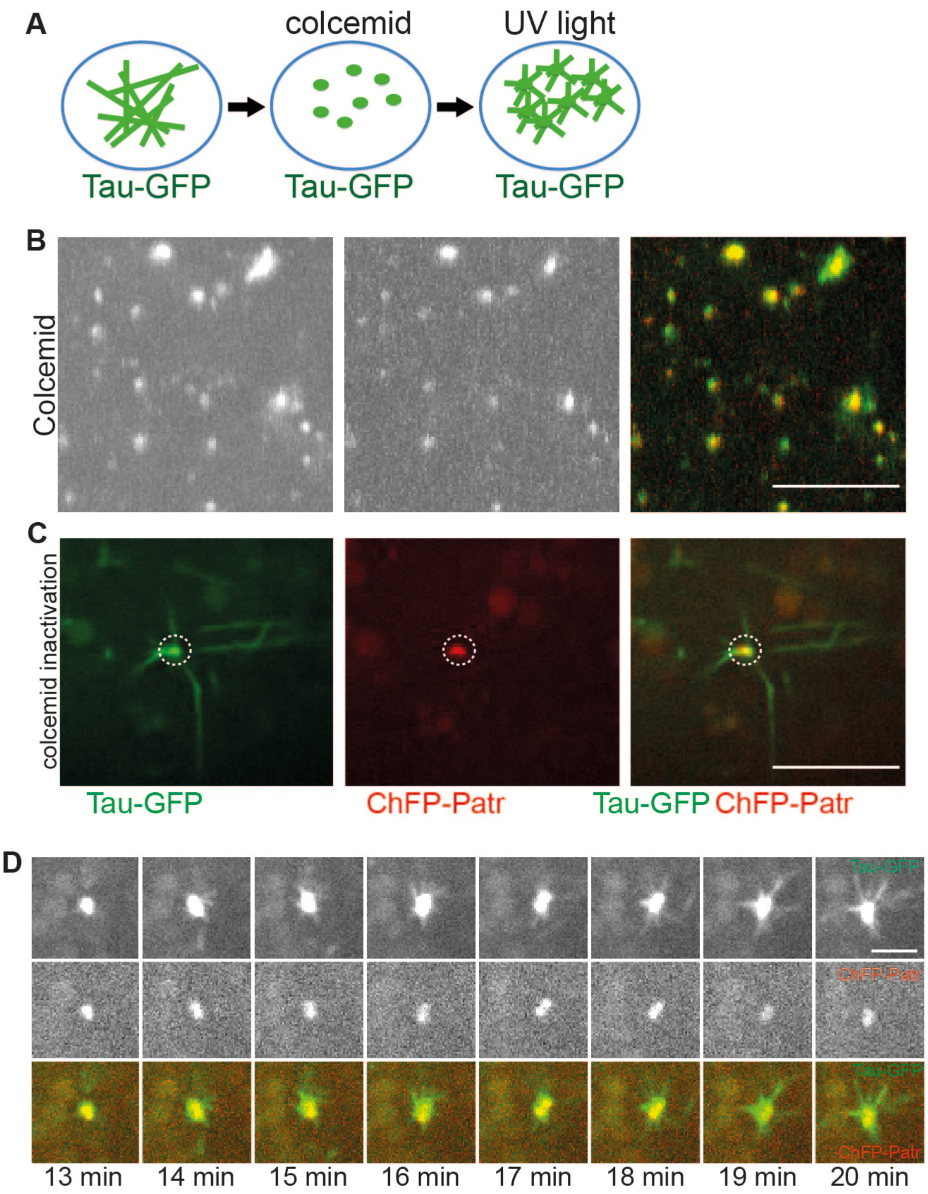

Figure S1. Patronin foci are cortical noncentrosomal MTOCs.
Related to Figure 4.
(A) Diagram of the microtubule re-growth assay.
(B-C) A close-up of a region of the lateral cortex of an oocyte expressing Tau-GFP from the maternal α4tubulin promoter and UAS-Cherry-Patronin driven by nanos>Gal4.
(B) Patronin foci co-localise with the microtubule-binding protein Tau-GFP in the presence of the microtubule-depolymerising drug, colcemid.
(C) After colcemid inactivation with UV light, Tau-GFP labelled microtubules re-grow from the Cherry-Patronin foci, indicating that the latter represent MTOCs.
(D) Still images from Movie S5 showing an example of an active Cherry-Patronin MTOC after colcemid inactivation. The time in minutes since colcemid inactivation is shown at the bottom. Scale bars represent 5 μm.

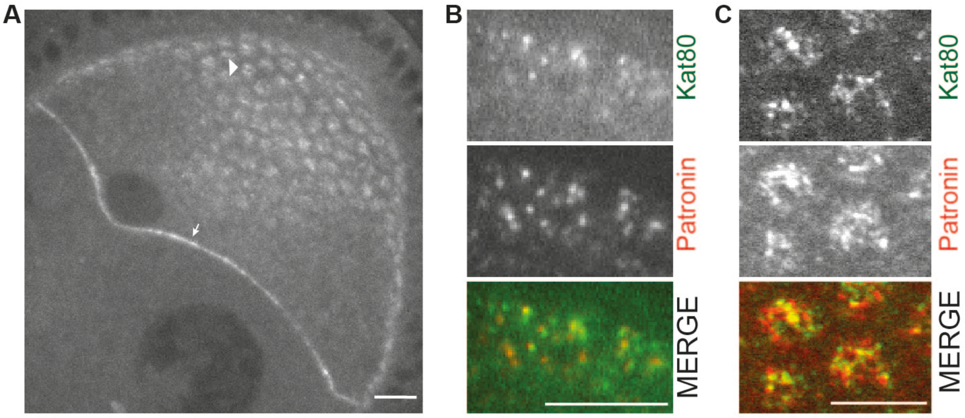

Figure S2. A protein trap insertion in Katanin 80 co-localises with Patronin ncMTOCs. Related to Figure 6.
(A) Katanin 80-YFP localises to the oocyte anterior and to the apical cortex of the follicle cells. The arrow points to the anterior and the arrowhead indicates the apical side of a follicle cell.
(B) Katanin 80 co-localises with Patronin in foci at the cortex of the oocyte.
(C) Katanin 80 and Patronin also co-localise at the apical side of the follicle cells.
Scale bars 10 μm.

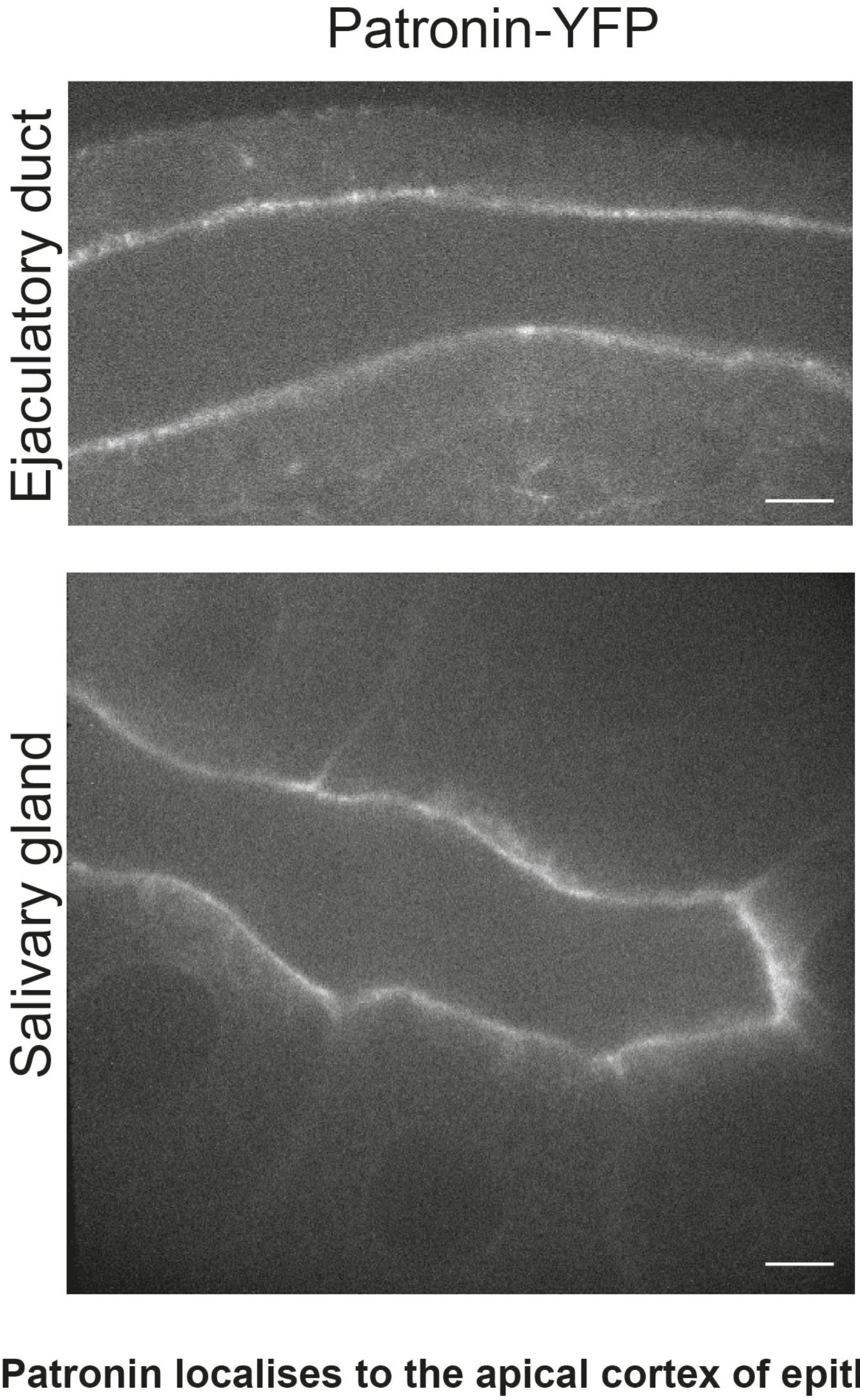

Figure S3. Patronin localises to the apical cortex of epithelial cells. Related to Figure 7.
Images of Patronin-YFP in the ejaculatory duct and in the larval salivary gland. Scale bars 10 μm.

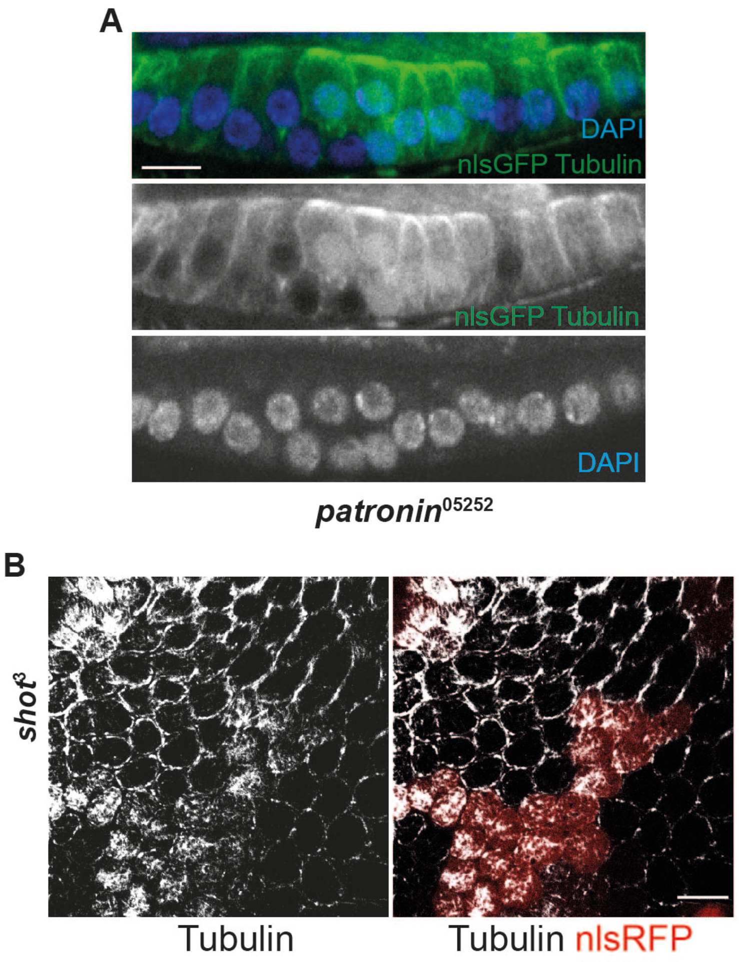

Figure S4. *patronin* and *shot* mutants disrupt the follicle cell epithelial monolayer. Related to Figure 7.
(A) *patronin*^{05252} mutant cells marked by the loss of nlsGFP lead to a disruption of epithelial organisation.
(B) A top view of the apical surface of the follicular epithelium containing a *shot*^{3} mutant clone marked by the loss of nlsRFP. The mutant cells lose the apical enrichment of microtubules that is visible in the adjacent wild-type cells.
Scale bars 10 μm.

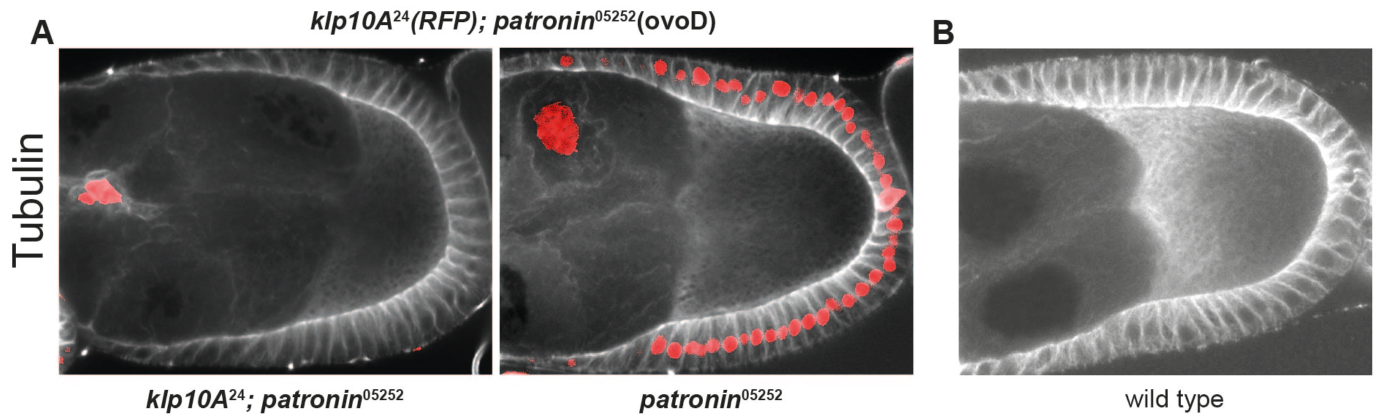

Figure S5. Removal of KLP10A does not rescue the *patronin*^{05252} microtubule phenotype in the oocyte. Related to Figure 7G.

(A) Germline clones mutant for both *klp10a* and *patronin*^{05252} (left) or *patronin*^{05252} only (right) stained with anti-tubulin. The *patronin*^{05252} clones were generated using the ovoD system, and *klp10a* germline clones were marked by the absence of nlsRFP in the nurse cells (left panel only; the red nuclei are heterozygous border cells).

(B) A wild type oocyte stained with anti-tubulin.

Supplemental Experimental Procedures

Fly stocks and genetics. *white*^{1118} was used as a wild-type stock throughout. The following mutant alleles and transgenic lines were used: *shot*^{2A2} (Chang et al., 2011), *shot*^{3} (Roper and Brown, 2003)(gift from N. Brown, University of Cambridge, UK), *patronin*^{EY05252} (Kyoto: 114436), *klp10A*^{24} (Radford et al., 2012) (gift from K. McKim, Waksman Institute and the State University of New Jersey, USA), *par-1*^{w3}/*par-1*^{6323} (Shulman et al., 2000), a protein trap line insertion in Jupiter-eGFP(Morin et al., 2001), maternal a4tubulin>GFP-Staufen(Martin et al., 2003), maternal a4tubulin>Tau-GFP(Doerflinger et al., 2003), UASp>GFP-EB1(Zhao et al., 2012), UASp>GFP-Par-1^{T786A} (Doerflinger et al., 2010), ncd>g-Tubulin37C-GFP (gift from S. Endow, Duke University, USA), UASp>Asl-GFP and UASp>Ana2-GFP(Stevens et al., 2010) (gift from J. Raff, University of Oxford, UK), ubi>EB1-GFP(Shimada et al., 2006), a protein trap insertion in Katanin80-YFP(Lowe et al., 2014). The mat-a4tub>Gal4-VP16 and nanos>Gal4 drivers were used to express UAS constructs in the germ line. Clones were generated with FRT G13, FRT G13 ovoD, FRT G13 RFP, FRT G13 GFP, FRT 19A RFP (Bloomington Stock Center) using the heat shock Flp/FRT system(Chou and Perrimon, 1992). The lethality and mutant phenotypes of *shot*^{2A2}/*shot*^{3} were rescued by a *shot* genomic BAC construct.

Co-immunoprecipitation. Ovarian extracts were prepared from wild-type, Shot-YFP and Kat80-YFP transgenic flies by dissecting ovaries (20-25 flies per genotype) into Schneider medium (Sigma) supplemented with 2.5% calf serum, followed by homogenization in ice cold PBS supplemented with 0.5% NP40, Complete Protease Inhibitor cocktail (Roche). The lipid-free fraction of the supernatant was collected after centrifugation at 16 000 g for 10 min at 4 ºC. Shot-YFP and Katanin 80-YFP were purified from 500 µL of ovarian extract using 5 µL of GFP-Trap®_MA (Chromotek) magnetic beads for 1 hr at 4 ºC with rotation. Input (4% of the extract) and bound fractions were analysed by SDS-PAGE and western blotting, using NuPage, 3-8% Tris-Acetate gels (ThermoFisher). Patronin was detected using 1:10000 rabbit anti-Patronin antibody(Goodwin and Vale, 2010) (gift from R. Vale, HHMI and UCSF, USA) and the ECL prime detection kit (GE LifeSciences).

SUPLEMENTAL REFERENCES

Chou, T.B., and Perrimon, N. (1992). Use of a yeast site-specific recombinase to produce female germline chimeras in Drosophila. Genetics *131*, 643–653.

Doerflinger, H., Benton, R., Shulman, J., and Johnston, D. (2003). The role of PAR-1 in regulating the polarised microtubule cytoskeleton in the Drosophila follicular epithelium. Development *130*, 3965.

Martin, S.G., Leclerc, V., Smith-Litière, K., and St Johnston, D. (2003). The identification of novel genes required for Drosophila anteroposterior axis formation in a germline clone screen using GFP-Staufen. Development *130*, 4201–4215.

Morin, X., Daneman, R., Zavortink, M., and Chia, W. (2001). A protein trap strategy to detect GFP-tagged proteins expressed from their endogenous loci in Drosophila. Proc Natl Acad Sci USA *98*, 15050–15055.

Radford, S.J., Harrison, A.M., and McKim, K.S. (2012). Microtubule-Depolymerizing Kinesin KLP10A Restricts the Length of the Acentrosomal Meiotic Spindle in Drosophila Females. Genetics *192*, 431–440.

Shimada, Y., Yonemura, S., Ohkura, H., Strutt, D., and Uemura, T. (2006). Polarized transport of Frizzled along the planar microtubule arrays in Drosophila wing

epithelium. Dev Cell *10*, 209–222.

Zhao, T., Graham, O.S., Raposo, A., and St Johnston, D. (2012). Growing microtubules push the oocyte nucleus to polarize the Drosophila dorsal-ventral axis. Science *336*, 999–1003.

SUPPLEMENTAL MOVIES

Movie S1 A time-lapse video of the microtubule-associated protein Jupiter-GFP in a wild-type stage 9 oocyte, showing the anterior-posterior gradient of microtubules. Related to Figure 1E.
Images were collected every 20 seconds on a spinning disc confocal microscope. The video is shown at 20 frames/sec.

Movie S2 A time-lapse video of Jupiter-GFP in a *shot*^{2A2} mutant stage 9 oocyte. Related to Figure 1E.
The microtubules are not enriched anteriorly and distribute more evenly throughout the oocyte. Images were collected every 30 seconds. The video is shown at 13.3 frames/sec.

Movie S3 Cherry-Patronin localises to the anterior/lateral cortex of wild-type oocytes (left) and throughout the cytoplasm in *shot*^{2A2} mutant oocytes (right). Related to Figure 3D. Images were collected every 10 seconds. The video is shown at 15 frames/s.

Movie S4 Microtubules grow from Cherry-Patronin foci after colcemid inactivation. Related to Figure 4C.
The growing plus ends of microtubules are labelled with EB1-GFP. The left panel shows EB1-GFP and the right panel Cherry-Patronin. The first 10 frames were taken before colcemid inactivation by a pulse of UV light. Images were collected every 3 seconds. The video is shown at 15 frames/s.

Movie S5 Microtubules grow from Cherry-Patronin foci after colcemid inactivation. Related to Figure 4C and Figure S1.
Microtubules are labelled with Tau-GFP. Images were collected every 1 minute. The video is shown at 10 frames/s.

Movie S6 Cherry-Patronin foci act as a source of growing MTs under steady state conditions in the absence of colcemid. Related to Figure 4D.
Microtubules are labelled with EB1-GFP. Cherry-Patronin is pseudocoloured in green. Images were collected every 1.6 seconds. The video is shown at 20 frames/s.

Movie S7 Mislocalisation of ncMTOCs labelled by EB1-GFP in *shot*^{2A2}. Related to Figure 4F.
Wild-type (left), *shot* (right). The first 10 frames were taken before colcemid inactivation by a pulse of UV light. Images were collected every second. The video is shown at 15 frames/s.

Movie S8 Microtubules labelled by EB1-GFP grow from the few ncMTOCs that form in *patronin*^{05252} mutant oocytes. Related to Figure 5C.
Wild type (left), *patronin*^{05252} (right). The first 4 frames were taken before colcemid inactivation by a pulse of UV light. Images were collected every second. The video is shown at 15 frames/s.

Movie S9 Microtubules grow from the apical cortex of the follicle cells where Cherry-Patronin foci (red) are localised. Related to Figure 7C.
Microtubules are labelled with EB1-GFP and Jupiter-GFP. Images were collected every 1.3 seconds. The video is shown at 30 frames/s.
