# Supplementary figures and images for: Patronin/Shot Cortical Foci Assemble the Noncentrosomal Microtubule Array that Specifies the Drosophila Anterior-Posterior Axis
Source: Dev Cell. 2016 Jul 11;38(1):61–72. doi: 10.1016/j.devcel.2016.06.010 (PMC4943857; doi:10.1016/j.devcel.2016.06.010)

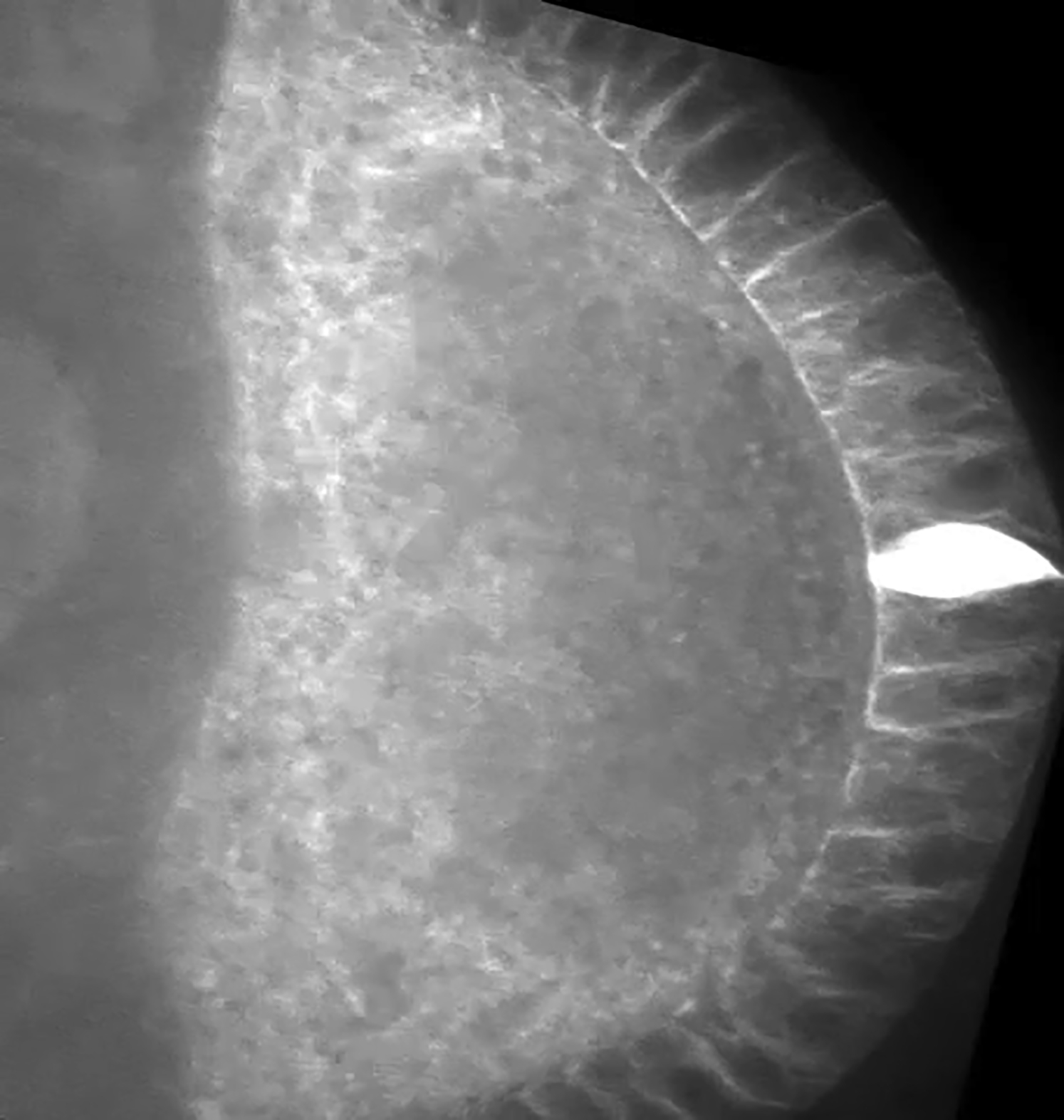

Supplement: Movie S1. Time-Lapse Video of the Microtubule-Associated Protein Jupiter-GFP in a Wild-Type Stage 9 Oocyte, Showing the Anterior-Posterior Gradient of Microtubules, Related to Figure 1E [file mmc2.jpg]

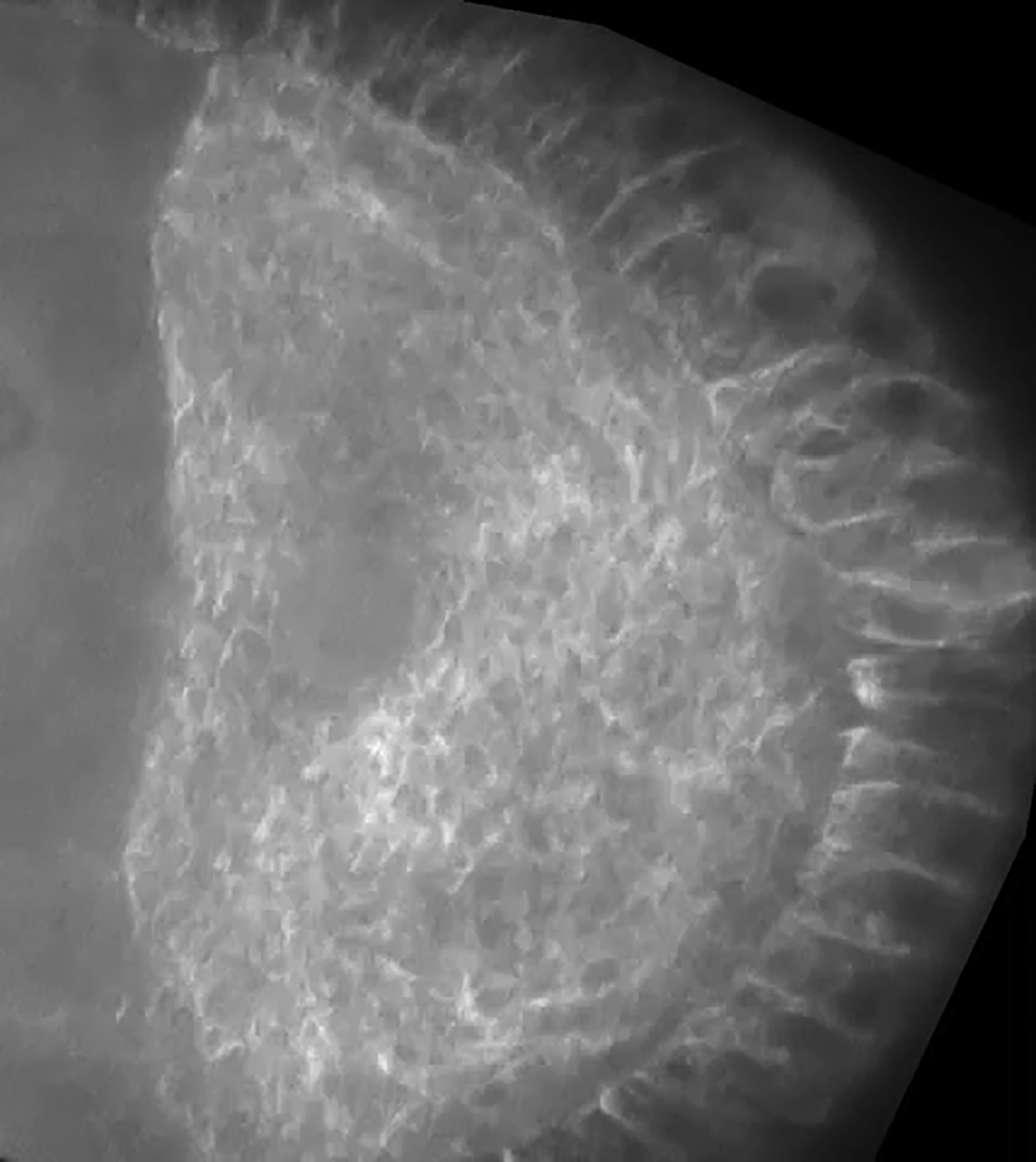

Supplement: Movie S2. Time-Lapse Video of Jupiter-GFP in a shot2A2 Mutant Stage 9 Oocyte, Related to Figure 1E [file mmc3.jpg]

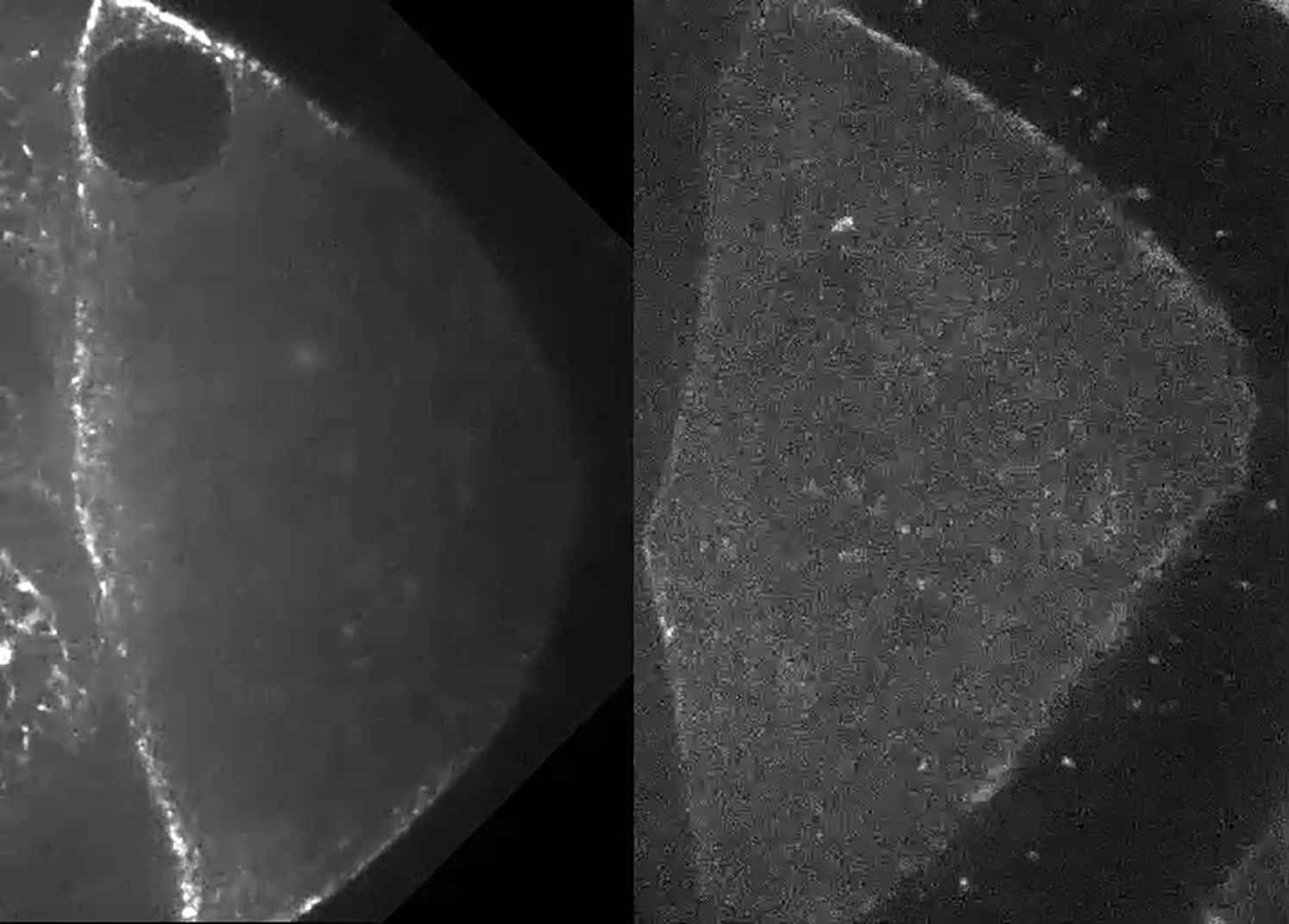

Supplement: Movie S3. Cherry-Patronin Localizes to the Anterior/Lateral Cortex of Wild-Type Oocytes, on the Left, and throughout the Cytoplasm in shot2A2 Mutant Oocytes, on the Right, Related to Figure 3D [file mmc4.jpg]

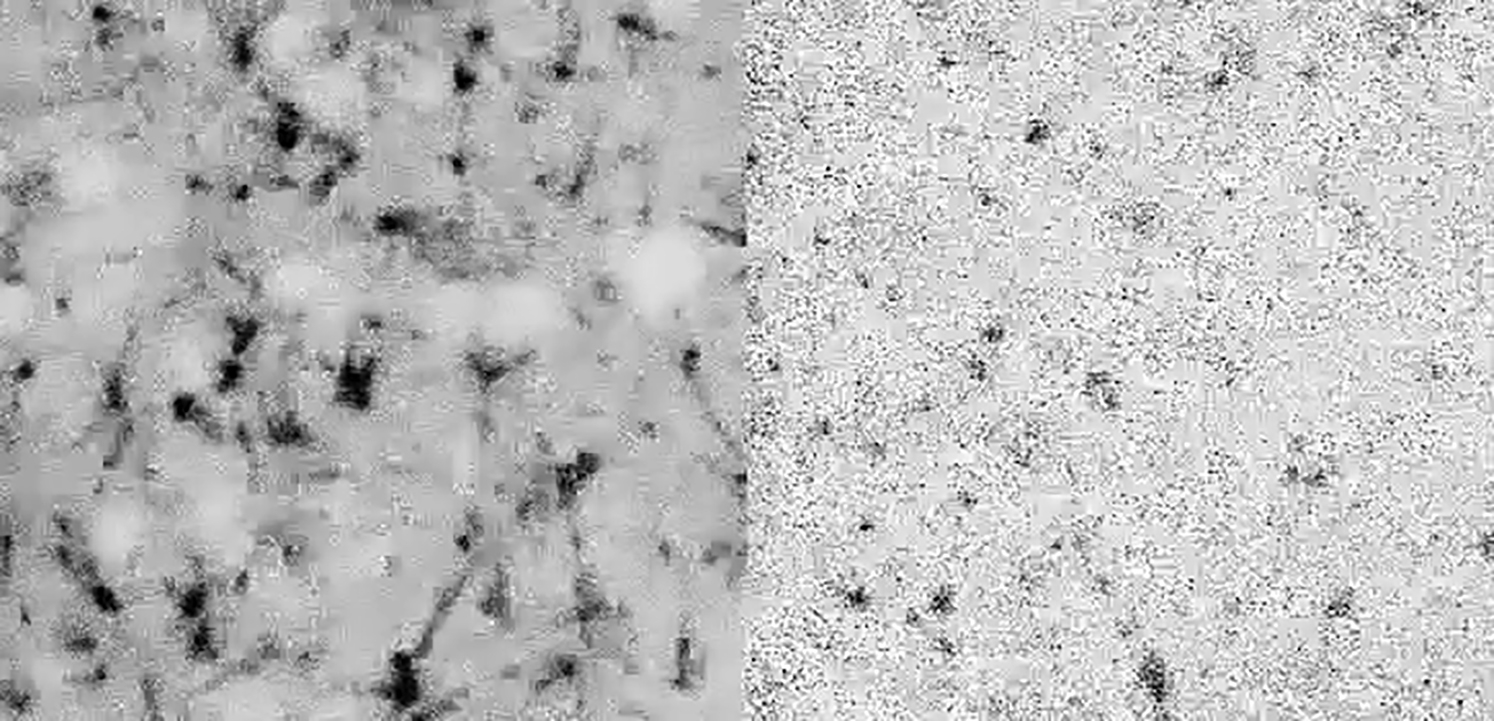

Supplement: Movie S4. Microtubules Grow from Cherry-Patronin Foci after Colcemid Inactivation, Related to Figure 4C [file mmc5.jpg]

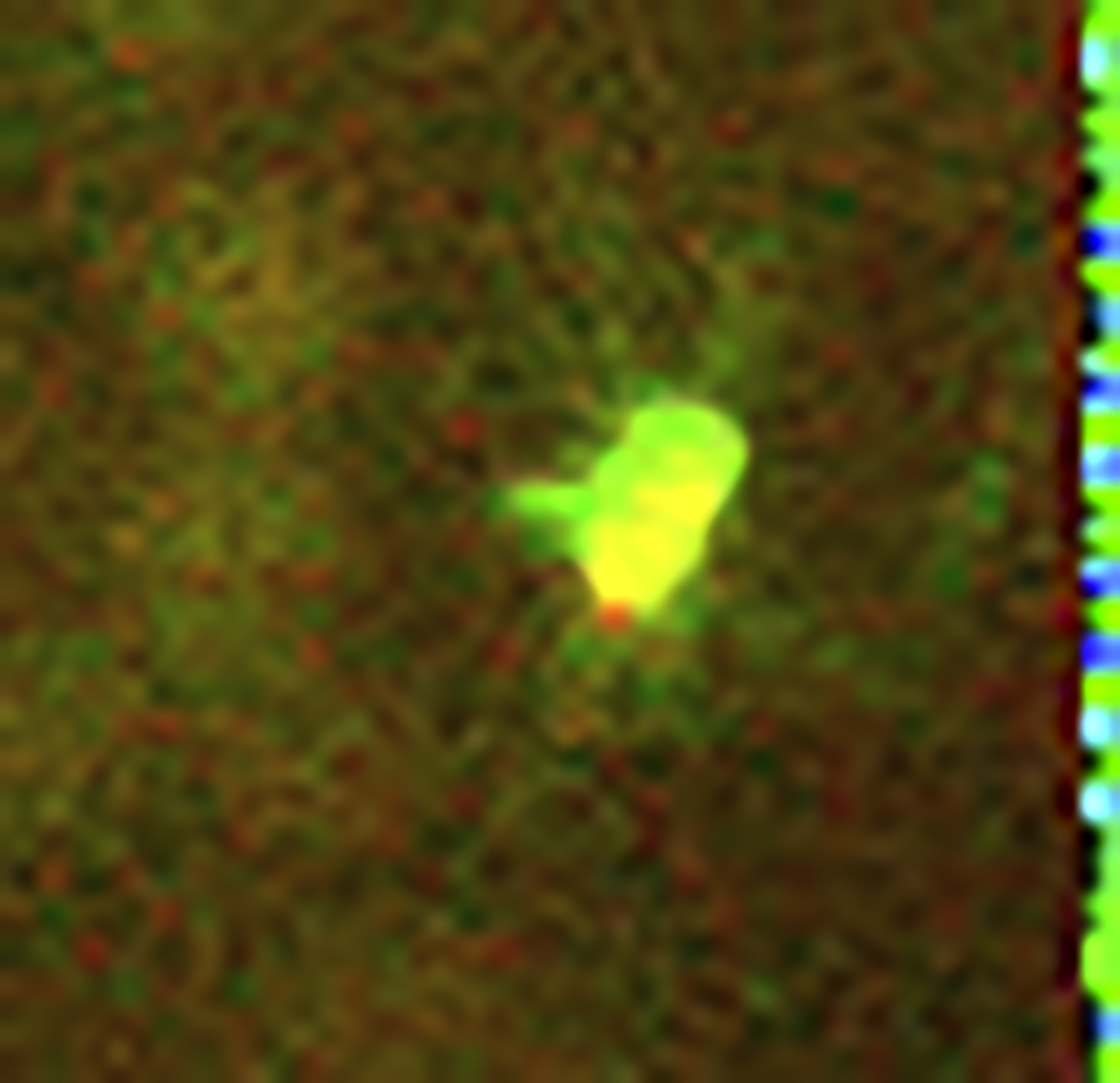

Supplement: Movie S5. Microtubules Grow from Cherry-Patronin Foci after Colcemid Inactivation, Related to Figures 4C and S1 [file mmc6.jpg]

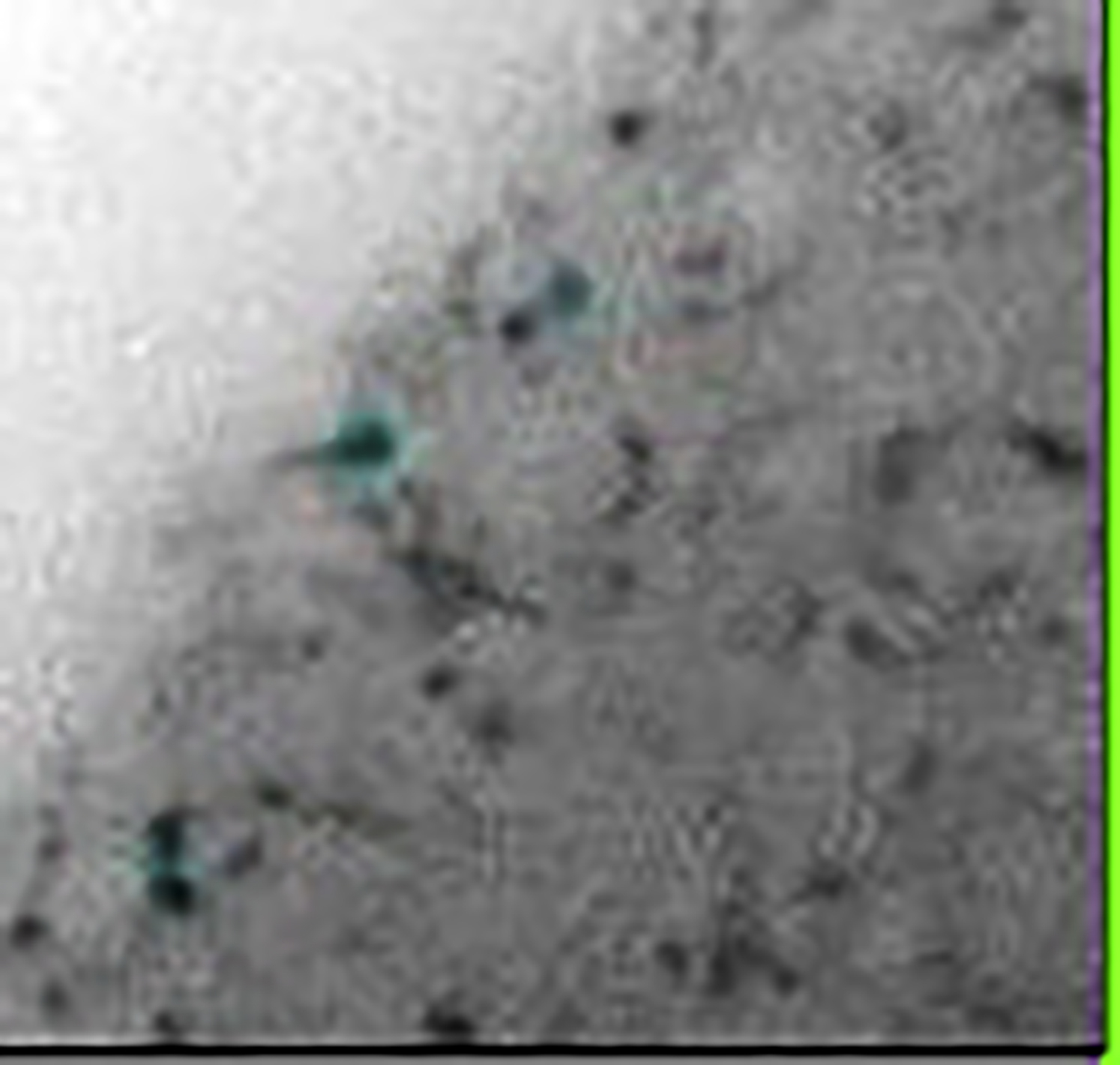

Supplement: Movie S6. Cherry-Patronin Foci Act as a Source of Growing MTs under Steady-State Conditions in the Absence of Colcemid, Related to Figure 4D [file mmc7.jpg]

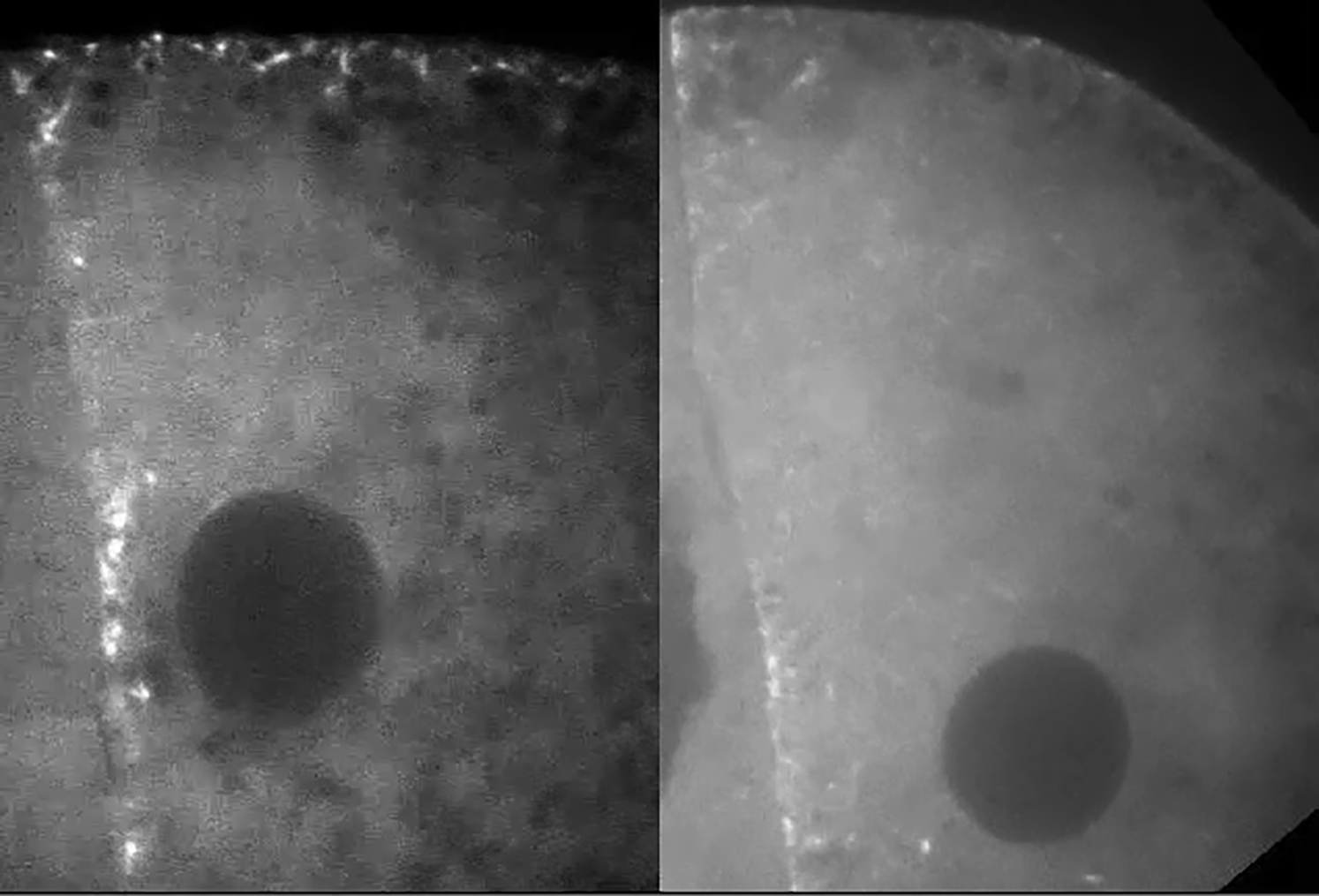

Supplement: Movie S7. Mislocalization of ncMTOCs Labeled by EB1-GFP in shot2A2, Related to Figure 4F [file mmc8.jpg]

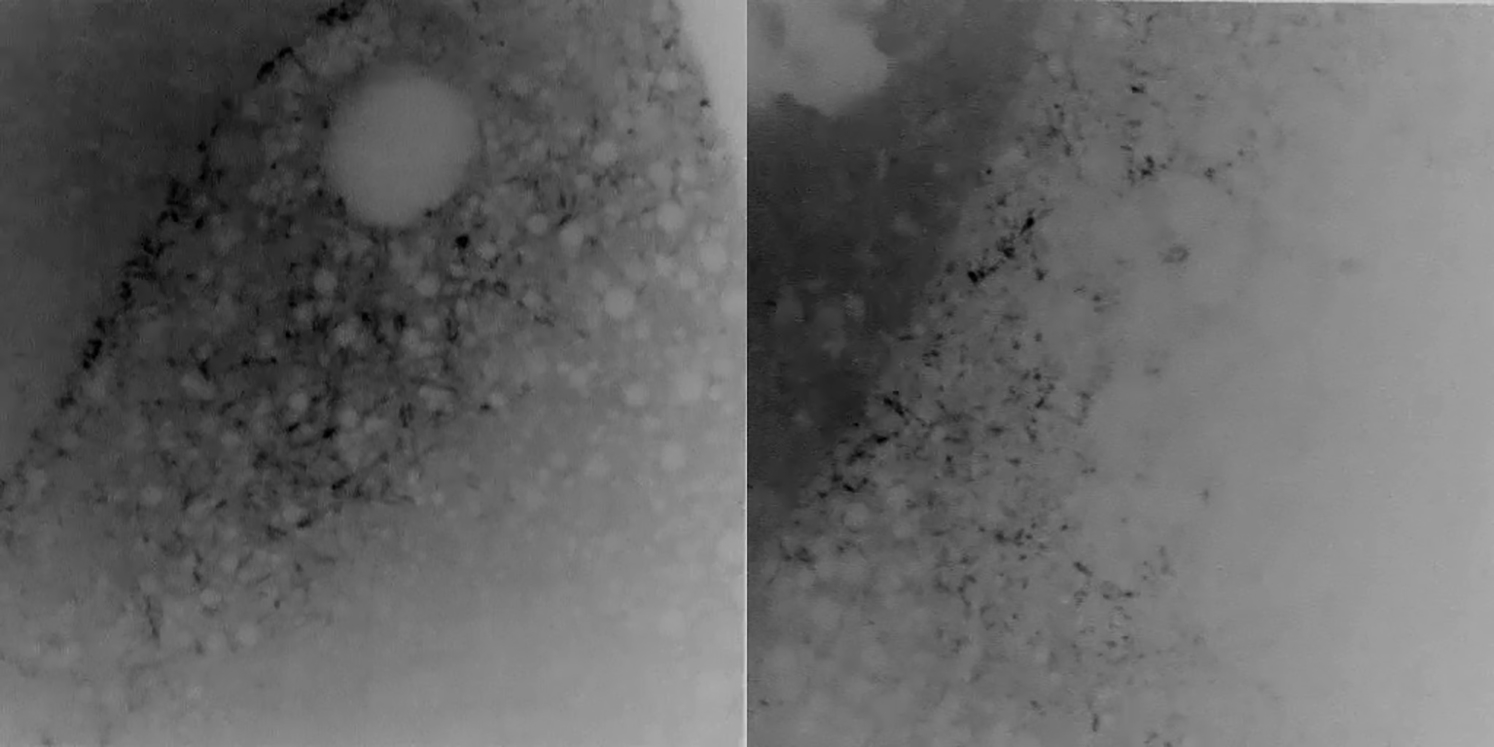

Supplement: Movie S8. Microtubules Labeled by EB1-GFP Grow from the Few ncMTOCs that Form in patronin05252 Mutant Oocytes, Related to Figure 5C [file mmc9.jpg]

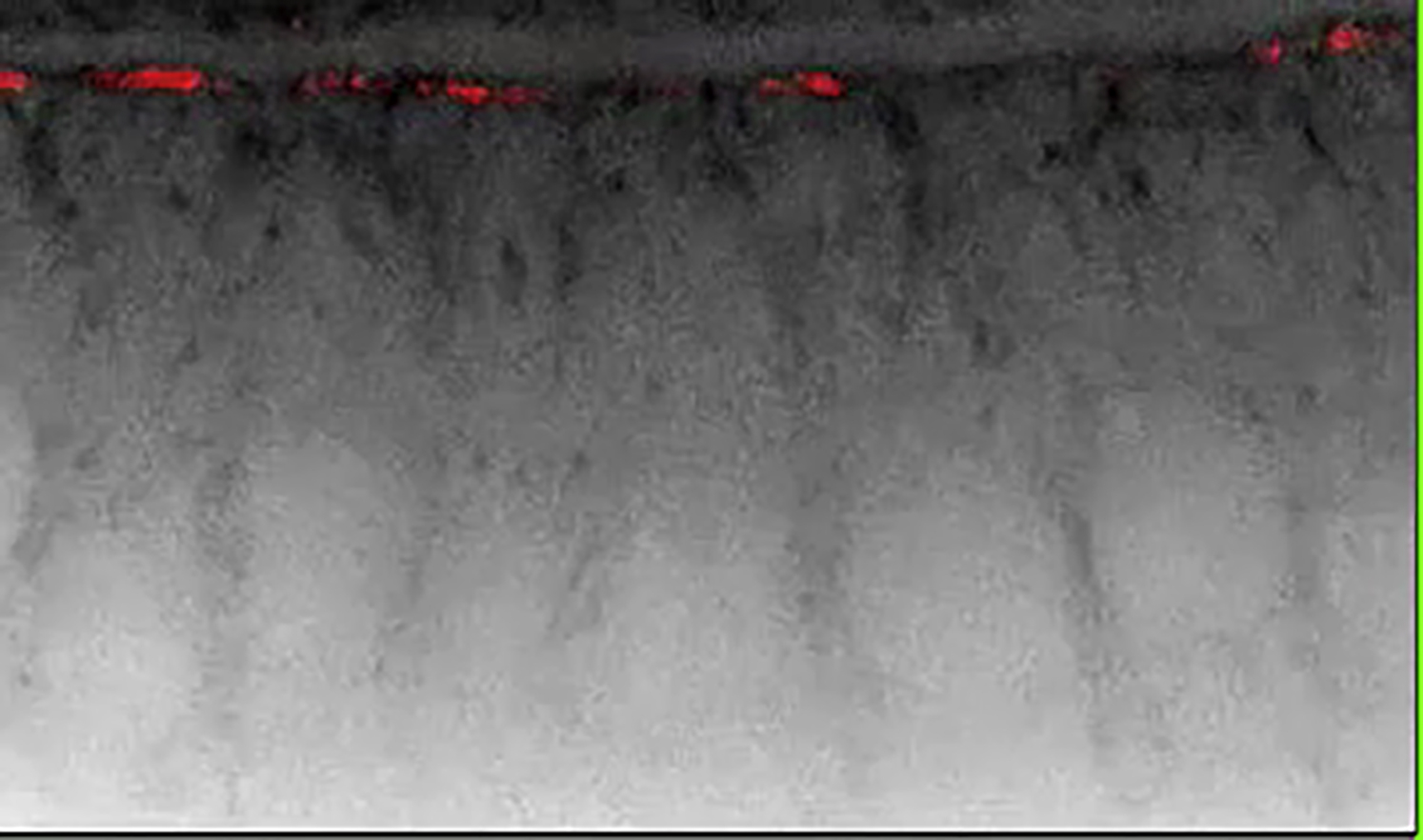

Supplement: Movie S9. Microtubules Grow from the Apical Cortex of the Follicle Cells where Cherry-Patronin Foci, in Red, Are Localized, Related to Figure 7C [file mmc10.jpg]
