# Supplementary material for: Patronin/Shot Cortical Foci Assemble the Noncentrosomal Microtubule Array that Specifies the Drosophila Anterior-Posterior Axis
Source: Dev Cell. 2016 Jul 11;38(1):61–72. doi: 10.1016/j.devcel.2016.06.010 (PMC4943857; doi:10.1016/j.devcel.2016.06.010)
Supplement: Document S2. Article plus Supplemental Information [file mmc11.pdf]

Developmental Cell

Article

Patronin/Shot Cortical Foci Assemble the Noncentrosomal Microtubule Array that Specifies the *Drosophila* Anterior-Posterior Axis

Graphical Abstract

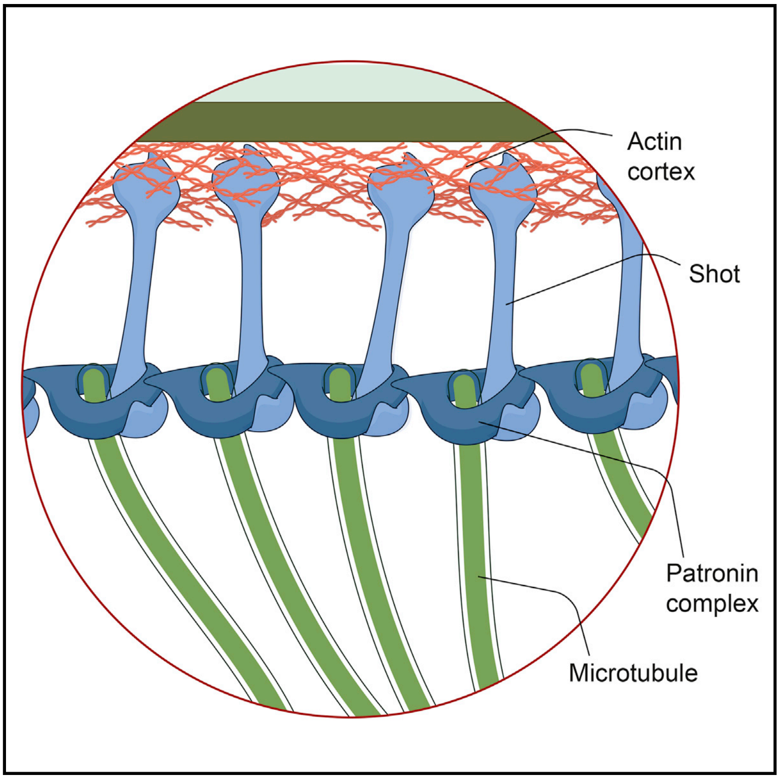

Highlights

- The *Drosophila* spectraplakin, Shot, recruits Patronin to form noncentrosomal MTOCs
- The actin-binding domain of Shot anchors the ncMTOCs to the oocyte cortex
- Par-1 excludes Shot from the posterior cortex to define the anterior-posterior axis
- Shot/Patronin ncMTOCs lack γ-tubulin and grow MTs from stabilized minus-end stumps

Authors

Dmitry Nashchekin,
Artur Ribeiro Fernandes,
Daniel St Johnston

Correspondence

d.stjohnston@gurdon.cam.ac.uk

In Brief

Many differentiated cell types lack centrosomes but still form highly polarized microtubule networks. Nashchekin et al. describe how the spectraplakin Shot and the microtubule minus-end-binding protein Patronin form a cortical noncentrosomal microtubule organizing center that acts a source of growing microtubules independently of γ-tubulin.

Nashchekin et al., 2016, Developmental Cell *38*, 61–72
July 11, 2016 © 2016 The Authors. Published by Elsevier Inc.
http://dx.doi.org/10.1016/j.devcel.2016.06.010

Developmental Cell
Article

CellPress

Patronin/Shot Cortical Foci Assemble the Noncentrosomal Microtubule Array that Specifies the *Drosophila* Anterior-Posterior Axis

Dmitry Nashchekin,[1] Artur Ribeiro Fernandes,[1] and Daniel St Johnston[1,*]
[1]The Gurdon Institute and the Department of Genetics, the University of Cambridge, Cambridge CB2 1QN, UK
*Correspondence: d.stjohnston@gurdon.cam.ac.uk
http://dx.doi.org/10.1016/j.devcel.2016.06.010

SUMMARY

Noncentrosomal microtubules play an important role in polarizing differentiated cells, but little is known about how these microtubules are organized. Here we identify the spectraplakin, Short stop (Shot), as the cortical anchor for noncentrosomal microtubule organizing centers (ncMTOCs) in the *Drosophila* oocyte. Shot interacts with the cortex through its actin-binding domain and recruits the microtubule minus-end-binding protein, Patronin, to form cortical ncMTOCs. Shot/Patronin foci do not co-localize with γ-tubulin, suggesting that they do not nucleate new microtubules. Instead, they capture and stabilize existing microtubule minus ends, which then template new microtubule growth. Shot/Patronin foci are excluded from the oocyte posterior by the Par-1 polarity kinase to generate the polarized microtubule network that localizes axis determinants. Both proteins also accumulate apically in epithelial cells, where they are required for the formation of apical-basal microtubule arrays. Thus, Shot/Patronin ncMTOCs may provide a general mechanism for organizing noncentrosomal microtubules in differentiated cells.

INTRODUCTION

Many differentiated animal cells and all plant cells lack functional centrosomes, yet form highly organized microtubule (MT) arrays that play essential roles in cell polarity, organization, and function (Bartolini and Gundersen, 2006). For example, both *Drosophila* and rodent hippocampal neurons develop normally without active centrosomes, with the latter extending and even regenerating axons independently of centrosomal MT nucleation (Nguyen et al., 2011; Stiess et al., 2010). Most *Drosophila* tissues lack functional centrosomes or microtubule organizing centers (MTOCs) in interphase (Rogers et al., 2008).

Anterior-posterior axis formation in the *Drosophila* oocyte provides a well-studied example of the role of noncentrosomal MTs. Although the oocyte contains centrosomes, which cluster near the nucleus, oogenesis proceeds normally in their absence (Basto et al., 2006; Januschke et al., 2006; Stevens et al., 2007). Instead, the majority of MTs grow from the anterior/lateral cortex, but not from the posterior, where the plus ends concentrate (Clark et al., 1994, 1997; Parton et al., 2011; Theurkauf et al., 1992). This noncentrosomal MT array directs the localization of *bicoid* and *oskar* mRNAs to the anterior and posterior poles of the oocyte, respectively, to define the main body axis of the embryo (St Johnston, 2005; Zimyanin et al., 2008). 3D modeling of the oocyte MT cytoskeleton has shown that restricting MT minus ends to the anterior/lateral cortex is sufficient to generate an MT network that can direct the transport of *oskar* mRNA to the oocyte posterior by kinesin (Khuc Trong et al., 2015).

The formation of this polarized MT array is under the control of the PAR proteins, which localize in mutually antagonistic anterior and posterior cortical domains (Doerflinger et al., 2010; Shulman et al., 2000). The posterior crescent of the Par-1 kinase transmits this cortical polarity to the MT cytoskeleton by excluding minus ends from the oocyte posterior. It is not known, however, how PAR-1 activity is transduced into the asymmetric organization of MT minus ends, nor how the minus ends associate with the anterior/lateral cortex.

The recent discovery of the Patronin family of MT minus-end-binding proteins, consisting of Patronin in *Drosophila*, CAMSAP1, 2, and 3 in mammals, and PTRN-1 in worms, has begun to reveal how the minus ends of noncentrosomal MTs are organized and maintained (Akhmanova and Steinmetz, 2015; Baines et al., 2009; Goodwin and Vale, 2010; Marcette et al., 2014; Meng et al., 2008; Richardson et al., 2014). The Patronins recognize and stabilize free MT minus ends by protecting them from depolymerization (Goodwin and Vale, 2010; Hendershott and Vale, 2014; Jiang et al., 2014). Patronins appear to play a particularly important role in organizing MTs in differentiated cells. CAMSAP3 localizes to the apical domain in epithelial cells, where it is required for the formation of the apical-basal array of MTs (Tanaka et al., 2012; Toya et al., 2016; Zheng et al., 2013). CAMSAP2 stabilizes neuronal MTs in axon and dendrites, and its knockdown leads to defects in axon specification and dendritic branch formation (Yau et al., 2014). Similarly, *Caenorhabditis elegans* PTRN-1 is required for normal neurite morphology and axon regeneration (Chuang et al., 2014; Marcette et al., 2014; Richardson et al., 2014). The function of *Drosophila* Patronin has only been examined in cultured S2 cells, where its depletion leads to a decrease in MT number and an increase in free moving MTs (Goodwin and Vale, 2010).

Although it is now clear that the Patronins play an important role in organizing noncentrosomal MTs in differentiated cells, little is known about the regulation of the distribution and activity of

This is an open access article under the CC BY license (http://creativecommons.org/licenses/by/4.0/).

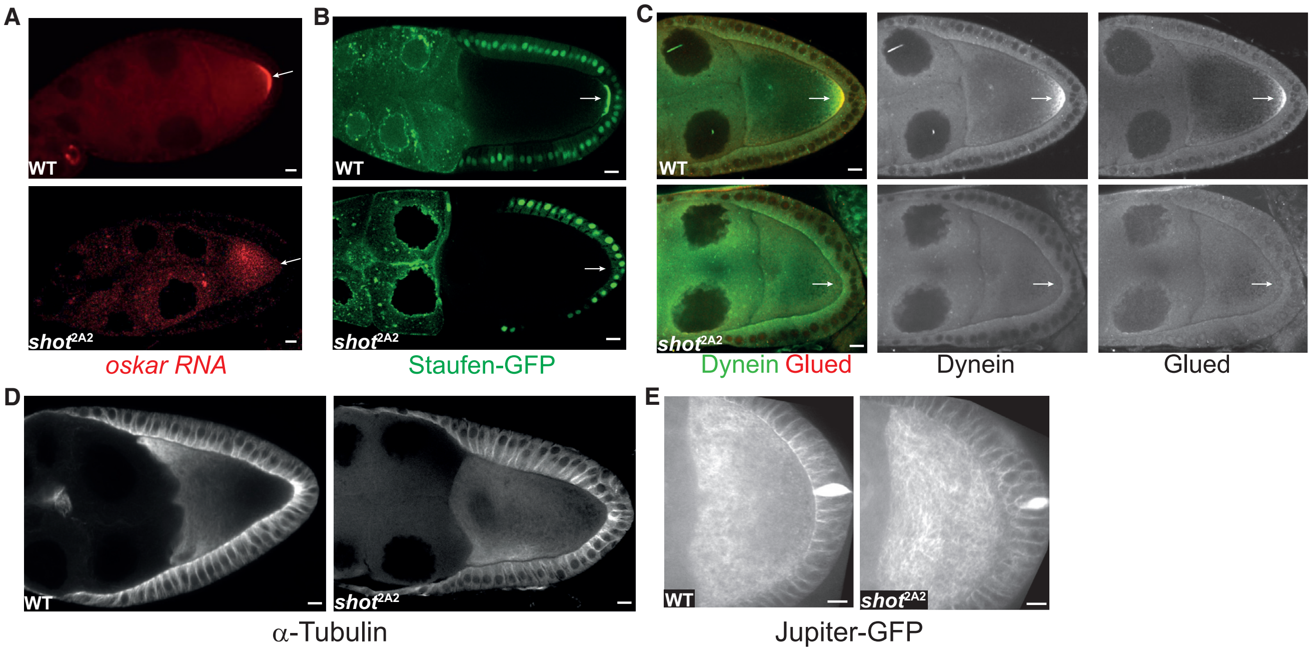

Figure 1. Shot Is Required for Oocyte Polarity and Microtubule Organization

(A–C) *oskar* mRNA (A), Staufen (B), Dynein, and Glued (C) localization in wild-type (WT; top) and *shot*^{2A2} mutant (bottom) oocytes. Arrows point to the oocyte posterior.

(D) MT organization detected by α-tubulin staining of WT (left) and *shot*^{2A2} mutant oocytes (right).

(E) Live imaging of Jupiter-GFP in WT (left) and *shot*^{2A2} mutant oocytes (right). The images are stills from Movies S1 (WT) and S2 (*shot*^{2A2}).

Scale bars represent 10 μm.

the Patronins themselves. Here we show that Patronin is recruited to the anterior/lateral cortex of the *Drosophila* oocyte by the spectraplakin, Shot, under the control of Par-1. These Shot/Patronin complexes form the cortical noncentrosomal MTOCs that organize the polarized MT network in the oocyte, which specifies the anterior-posterior axis.

RESULTS

Shot Is Required for the Polarized Organization of MTs in the Oocyte

We previously isolated 11 new alleles of *short stop* (*shot*) in a screen for dominant suppressors of the bicaudal phenotype caused by mislocalizing *oskar* mRNA to the oocyte anterior (Chang et al., 2011). Shot is the single *Drosophila* spectraplakin, a giant cytoskeletal linker protein with an N-terminal actin-binding domain and two C-terminal domains that bind MT, the GAS2 domain, which binds to the MT lattice, and a more C-terminal domain that associates with MT plus ends through the +TIP, EB1 (Applewhite et al., 2010; Sun et al., 2001). Null alleles of *shot* block the specification of the oocyte, and this is also the case for 10 out of 11 of the new alleles (Roper and Brown, 2004). Some germline clones of *shot*^{2A2} are not blocked in oogenesis, however, and develop to later stages, occasionally laying fertilized eggs that develop into larvae that lack an abdomen. Since this is a typical posterior group phenotype, we examined whether the posterior determinant, *oskar* mRNA, is correctly localized in *shot*^{2A2} mutants. Both *oskar* RNA and Staufen-GFP (an RNA-binding protein associated with *oskar*) fail to localize to the oocyte posterior in *shot*^{2A2} germline clones (Figures 1A and 1B). To determine whether Shot is specifically required for *oskar* mRNA localization or plays a more general role in kinesin-dependent transport to the posterior, we also examined the localization of Dynein and the dynactin subunit, Glued, which are transported to the oocyte posterior by kinesin independently of *oskar* mRNA (Brendza et al., 2002; Palacios and St Johnston, 2002). Neither Dynein nor Glued are localized in *shot*^{2A2} oocytes, indicating that either kinesin activity is inhibited or the MT plus ends are not concentrated at the posterior pole (Figure 1C).

We next examined the overall organization of the MTs in fixed and living oocytes. Staining of fixed oocytes with anti-tubulin and in vivo labeling of MTs in living oocytes with Jupiter-GFP (Karpova et al., 2006) reveals the anterior-posterior gradient of MTs in wild-type with the highest concentration of MTs at the anterior (Figures 1D and 1E; Movie S1). This anterior enrichment is lost in *shot*^{2A2} and the MT organization becomes somewhat variable, with a much more even distribution throughout the oocyte cytoplasm (Figures 1D and 1E, right panels; Movie S2).

Par-1 Regulates the Association of the Shot Actin-Binding Domain with the Cortex

Shot localizes to the anterior and lateral cortex of the oocyte, but is absent from the posterior, following the predicted distribution of MT minus ends. Shot is also strongly enriched at the apical side of the epithelial follicle cells that surround the developing egg chamber (Figure 2A, left). YFP-tagged Shot expressed from a transgenic bacterial artificial chromosome (BAC) rescuing

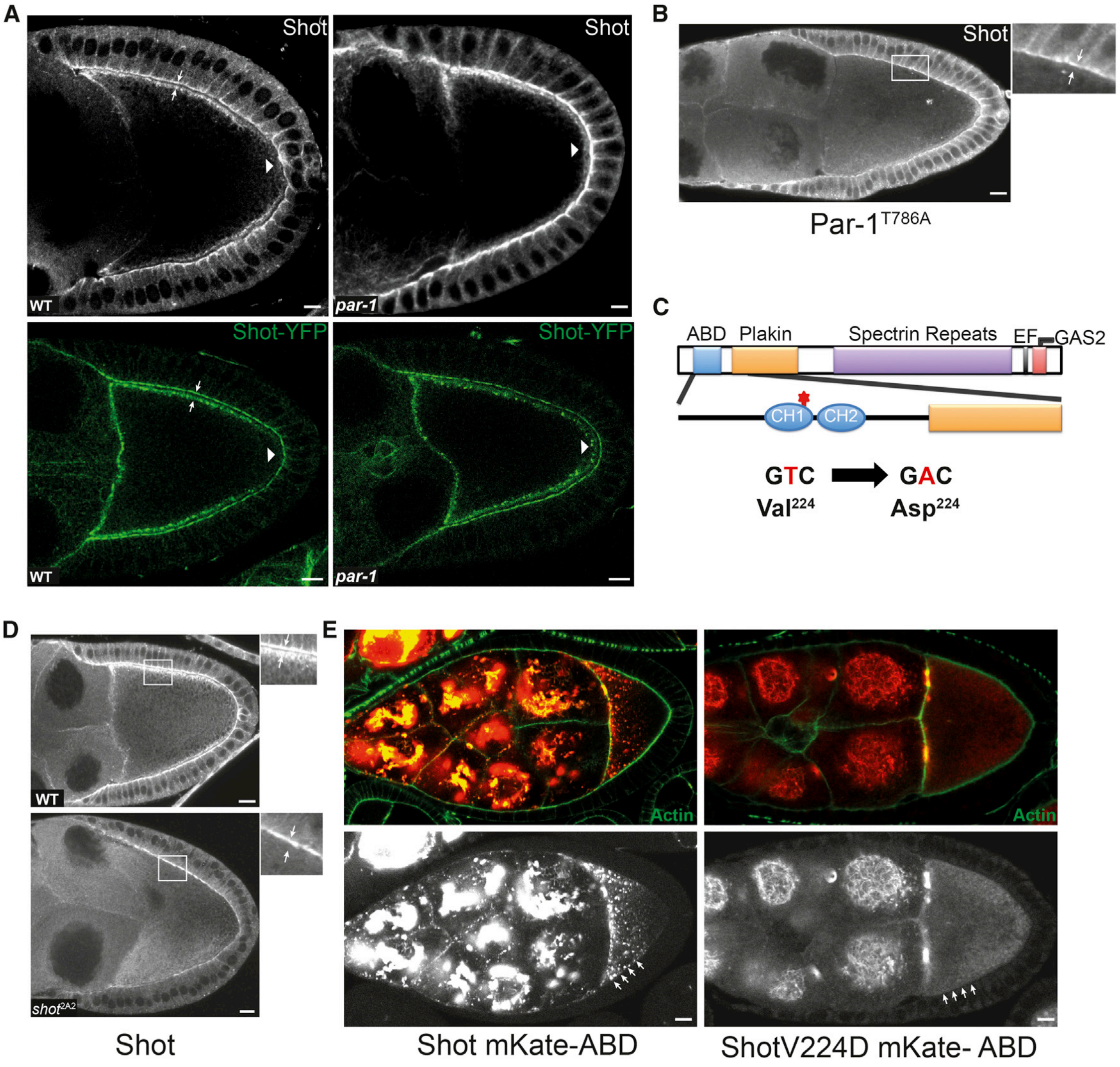

Figure 2. The Cortical Localization of Shot Depends on Its Actin-Binding Domain and Is Inhibited by Par-1

(A) Shot localizes to the anterior-lateral cortex and is excluded from the oocyte posterior (left). Shot spreads around the oocyte posterior in the *par-1*^{6323}/par-1^{W3} mutant (right). Top: Shot antibody. Bottom: Shot-YFP genomic BAC.

(B) Overexpression of Par-1^{T786A}-GFP displaces Shot from the oocyte cortex.

(C) Diagram of the domain structure of Shot, indicating the position and the nature of the point mutation in *shot*^{2A2}. CH, calponin homology domain. CH1 and CH2 form the actin-binding domain (ABD).

(D) *shot*^{2A2} disrupts the localization of Shot to the oocyte cortex. The small boxes on the right are higher-magnification views showing the localization of Shot to the lateral cortex of the wild-type (WT) oocyte and its absence in *shot*^{2A4}. Shot also localizes to the apical cortex of the follicle cells.

(E) Wild-type Shot ABD (left) localizes to the anterior-lateral cortex, whereas the Shot ABD with a Val224 to Asp mutation (right) does not.

Arrows point to the cortical Shot signal in the oocyte and to the underlying apical signal in the epithelial follicle cells (A, B, D). Arrowheads in (A) point to posterior. Arrows in (E) indicate the cortical signal. Scale bars represent 10 μm.

construct shows an identical distribution in both the follicle cells and oocyte. We therefore examined whether the interaction of Shot with the oocyte cortex is under the control of the cortical Par proteins that control the polarity of the MT cytoskeleton. In *par-1* mutant oocytes, MTs grow from the posterior cortex as well as the anterior/lateral cortex, and the MT cytoskeleton loses its asymmetry, whereas Par-1^{T786A}, which has a uniform cortical distribution, abolishes all MT growth from the cortex (Doerflinger et al., 2010; Parton et al., 2011). Shot responds to Par-1 activity in the same way as MTs: it extends around the posterior in the absence of Par-1, and is lost from the cortex in oocytes over-expressing Par-1^{T786A} (Figures 2A and 2B). Thus, Shot is down-stream of Par-1, consistent with it playing a role in MT minus-end localization.

Sequencing of *shot*^{2A2} reveals that it is a point mutation in the first calponin homology domain of the N-terminal actin-binding domain (ABD) of Shot, changing Val^{224} (isoform PE) to Asp (Figure 2C). Val^{224} is well conserved among ABD-containing proteins. Structural analysis of the interaction of fimbrin with F-actin showed that the equivalent to Val^{224} (Val^{212} in fimbrin) directly

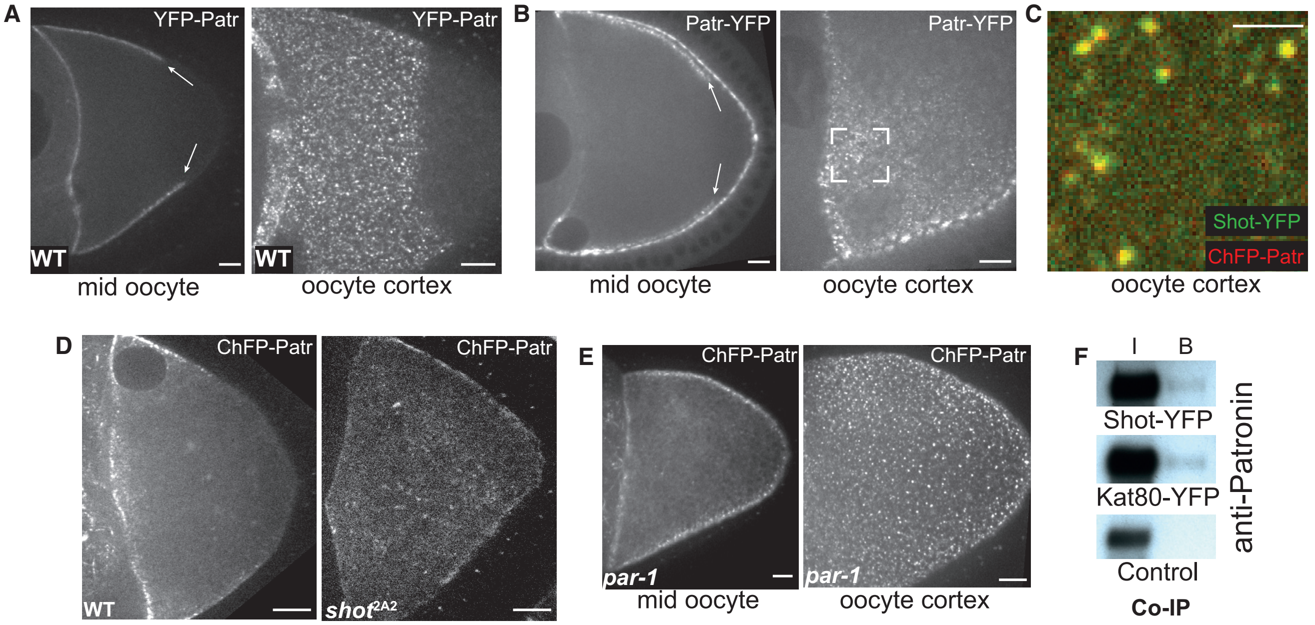

Figure 3. Patronin Is Recruited into Cortical Foci by Shot

(A and B) YFP-Patronin (expressed in the germline under the control of the maternal tubulin-α4 promoter) (A) and endogenously tagged Patronin-YFP (B) in living stage 9 oocytes. Patronin localizes to the anterior/lateral cortex of the oocyte, where it forms discrete foci. The right-hand panels are projections of several z sections spanning the oocyte cortex. The white rectangle in (B) marks a region where the oocyte cortex is in focus, showing the Patronin-YFP foci. The arrows point to the posterior boundary of the domain of Patronin foci in the oocyte. WT, wild-type.

(C) A close-up of a region of the lateral cortex of a living oocyte, showing the co-localization of Shot-YFP and Cherry-Patronin in cortical foci. UAS-Cherry-Patronin was expressed in the germline under the control of *nanos*-Gal4. Scale bar represents 2.5 μm.

(D) Cherry-Patronin localization in wild-type (WT; left) and *shot*^{2A2} mutant oocytes (right). UAS-Cherry-Patronin expression was driven by maternal α4tubulin-Gal4. These still images were taken from Movie S3.

(E) Cherry-Patronin foci extend around the oocyte posterior in *par-1*^{w3}/*par-1*^{6323} mutant oocytes (compare with A, B, and D). Images are projections of several z sections spanning the oocyte cortex.

(F) Co-immunoprecipitation (IP) of Patronin by Shot-YFP and Katanin 80-YFP. I, input. B, bound.

Scale bars represent 10 μm, except in (C). See also Figure S2.

contacts F-actin (Hanein et al., 1998). In agreement with this, Shot loses its association with the actin-rich cortex in *shot*^{2A2} and is mainly cytoplasmic (Figure 2D). Like full-length Shot, the Shot ABD is enriched at the anterior-lateral cortex (Figure 2E, left). Introducing the Val^{224} to Asp mutation into the Shot ABD disrupts its cortical localization, although the protein still shows an enrichment at the ring canals, which is not observed with the full-length protein (Figure 2E, right). Thus, Shot is recruited to the cortex through its ABD, presumably by direct binding to cortical F-actin, and this interaction is inhibited at the posterior by Par-1.

Shot Recruits Patronin Foci to the Oocyte Cortex

We took advantage of the recent identification of Patronin/CAMSAP as an MT minus-end-binding protein to analyze the relationship between cortical Shot and the distribution of MT minus ends in the oocyte (Goodwin and Vale, 2010; Jiang et al., 2014). Live imaging of both transgenic and endogenously tagged Patronin reveals that it localizes to anterior/lateral cortex in the expected distribution of MT minus ends (Figures 3A and 3B; Movie S3, left panel). Importantly, Patronin co-localizes with Shot in distinct cortical foci (Figure 3C). Patronin localization is Shot dependent, as it becomes largely cytoplasmic in *shot*^{2A2} (Figure 3D and Movie S3, right panel). Furthermore, the cortical Patronin foci extend around the posterior cortex in *par-1* mutant oocytes, as Shot does, consistent with the two proteins being in the same complex (Figure 3E). In agreement with this, Patronin co-immunoprecipitates with Shot-YFP from ovary extracts (Figure 3F). The fact that both Patronin and Shot are no longer cortical in *shot*^{2A2} indicates that Shot anchors Patronin to the cortex, providing an explanation of how the asymmetric localization of Shot controls the polarized distribution of MT minus ends in the oocyte.

Patronin Cortical Foci Are Noncentrosomal MTOCs

It has previously been shown, using Tau-GFP to label MTs and EB1-GFP to label the growing MT plus ends, that oocyte MTs grow out from noncentrosomal foci that can be visualized using an MT regrowth assay (Parton et al., 2011). Upon colcemid treatment, both proteins accumulate in cortical foci. Local inactivation of the colcemid with a pulse of UV light allows MTs to regrow from the cortex (Figure 4A). We therefore examined whether the MTs grow from the Patronin foci. Both EB1-GFP and Tau-GFP accumulate in the cortical Patronin foci upon colcemid treatment, indicating that these contain stable MT minus ends (Figures 4B and S1B). Furthermore, after colcemid inactivation

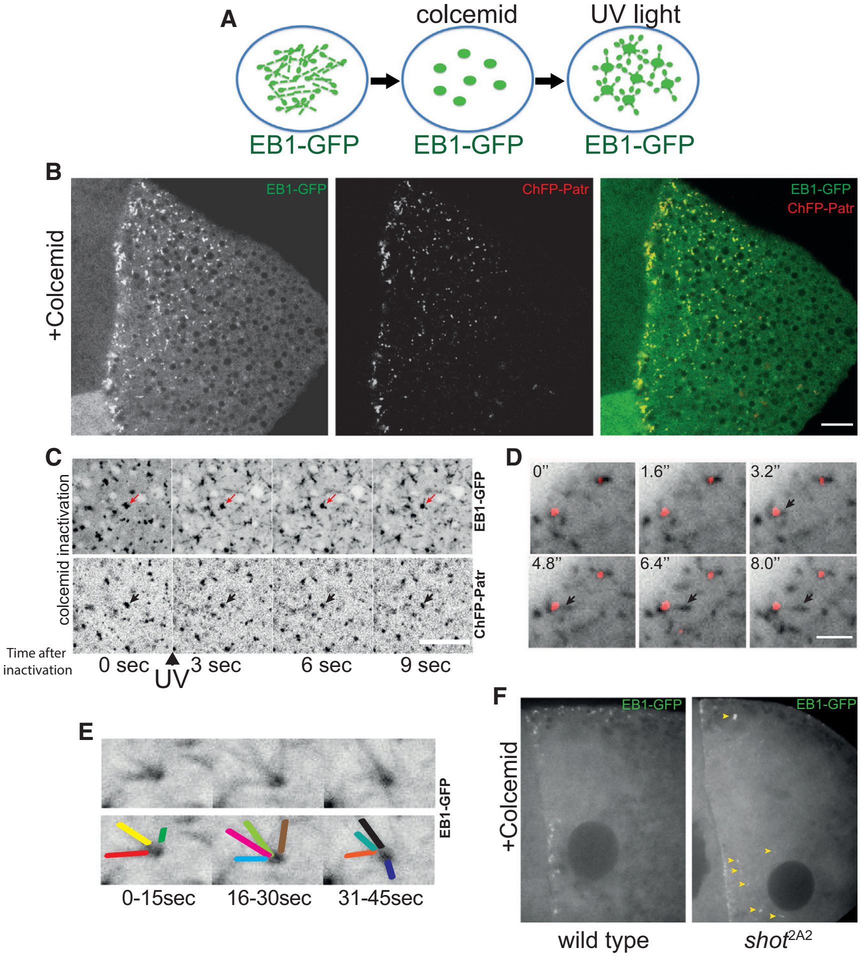

Figure 4. Patronin Foci Are Cortical Noncentrosomal MTOCs

(A) Diagram of the MT regrowth assay.

(B) Patronin foci co-localize with the MT plus-end marker EB1-GFP in the presence of colcemid. Scale bar represents 10 μm.

(C) Still images from Movie S4 showing new EB1-GFP comets growing out from the Patronin foci a few seconds after colcemid inactivation. The arrows indicate a single active MTOC in successive frames. Scale bar represents 10 μm.

(D) Patronin foci are active MTOCs that produce new MTs in the absence of colcemid. Images taken from Movie S6. The arrows point to a new EB1-GFP comet that marks the plus end of a microtubule growing from a Patronin MTOC (red). Scale bar represents 2 μm.

(E) A single Patronin focus produces many MTs that grow in multiple directions. The images are projections of several time points over 15-s intervals. Each colored line represents a new EB1-GFP track (bottom panel). Images taken from Movie S4.

(F) Localization of EB1-GFP foci in wild-type (left) and *shot*^{2A2} (right) oocytes after colcemid treatment. Images taken from Movie S7. Arrowheads point to the cytoplasmic ncMTOCs in the *shot*^{2A2} mutant.

with UV light, EB1-GFP and Tau-GFP label growing MTs that emerge from the Patronin foci (Figures 4C, S1C, and S1D; Movies S4 and S5). The Patronin foci also act as a source of growing MTs under steady-state conditions in the absence of colcemid (Figure 4D and Movie S6). After colcemid inactivation, each Patronin focus produces an average of 11.5 new MTs per minute (n = 15; SEM = 0.75), providing a source of MTs that grow in multiple directions (Figure 4E). Moreover, these foci are the only visible source of growing MTs at the oocyte cortex, strongly suggesting that they represent the noncentrosomal, cortical MT organizing centers (ncMTOCs) from which MTs grow to form the polarized cytoskeleton in the oocyte.

In *shot*^{2A2} mutant oocytes, many of the foci fail to be retained at the oocyte cortex and redistribute throughout the oocyte cytoplasm, consistent with the loss of most Shot and Patronin from the cortex in this mutant (Figure 4F and Movie S7). These cytoplasmic foci remain active, however, producing growing MTs after colcemid inactivation, explaining why the overall polarity of the MT network is disrupted (Movie S7).

Patronin Is Required for ncMTOC Formation

A *patronin* null mutant blocks oogenesis at an early stage. To test whether Patronin is required for the activity of the cortical ncMTOCs in the oocyte, we therefore used a hypomorphic allele, *patronin*^{05252}, which strongly reduces Patronin levels (Bellen et al., 2004). *patronin*^{05252} homozygous oocytes contain 90% fewer cortical EB1-GFP foci after colcemid treatment than wild-type, and the remaining foci also generally recruit less EB1-GFP (Figures 5A and 5B). Nevertheless, the Patronin foci that form are still active, acting as a source of growing MTs after colcemid inactivation (Figure 5C and Movie S8). The density of MTs is also significantly reduced in *patronin*^{05252} clones, as expected from the reduced number of cortical ncMTOCs (Figure 5D). Despite the dramatic reduction in MT number, there are still sufficient MTs to direct the localization of Staufen/*oskar* mRNA complexes to the oocyte posterior, although the levels of localization are reduced by >40% (Figures 5E and 5F).

Patronin ncMTOCs Do Not Co-localize with γ-Tubulin

To further investigate the nature of the Shot/Patronin noncentrosomal MTOCs, we asked whether they contain γ-tubulin as the source of new MTs. Antibody staining of oocytes for γ-tubulin label only the centrosomes adjacent to the oocyte nucleus, but overexpressed γ-tubulin 37C-GFP is also seen in weak foci along the anterior/lateral cortex (Januschke et al., 2006; Parton et al., 2011). We therefore co-expressed γ-tubulin-GFP and Cherry-Patronin to determine whether the two proteins co-localize (Figures 6A and 6A′). Patronin labels some of the nuclear-associated, γ-tubulin foci, which probably correspond to the active centrosomes. The cortical Patronin foci do not co-localize with the γ-tubulin-GFP foci, however, and Shot/Patronin ncMTOCs contain no detectable γ-tubulin. Since MTs start to grow out from Patronin foci within 1 s of colcemid inactivation,

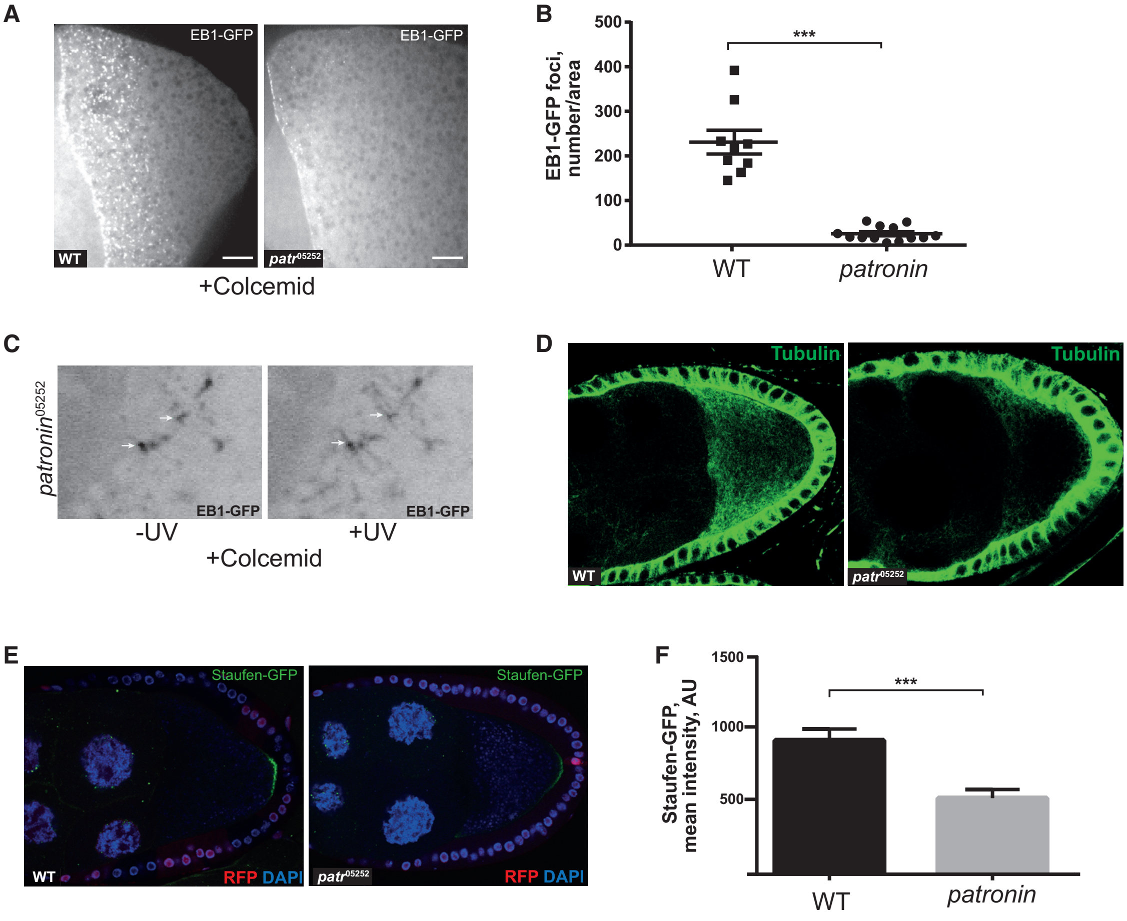

Figure 5. Patronin Is Required for the Formation of Cortical MTOCs

(A and B) The number of cortical MTOCs marked by EB1-GFP is reduced in *patronin*^{05252} mutant oocytes. (A) Images of wild-type (WT; left) and *patronin*^{05252} mutant (right) oocytes expressing nanos>UAS-EB1-GFP after colcemid treatment. The images are projections of the several z sections spanning the oocyte cortex. (B) Quantification of the number of cortical EB1-GFP foci after colcemid treatment in WT and *patronin*^{05252} oocytes. ***$p < 0.0001$. Error bars indicate the SEM.

(C) EB1-GFP foci before (left) and after (right) colcemid inactivation in a *patronin*^{05252} mutant oocyte. Close-up still images from Movie S8. The arrows indicate two of the activated MTOCs.

(D) MT density is strongly reduced in *patronin*^{05252} mutant oocytes. WT (left) and *patronin*^{05252} mutant (right) oocytes stained with anti-tubulin.

(E and F) Localization of Stau-GFP to the oocyte posterior is reduced in *patronin*^{05252} mutant oocytes. (E) Localization of Stau-GFP in WT (left) and *patronin*^{05252} (right) oocytes. *patronin*^{05252} germline clones were marked by the absence of nlsRFP. (F) Quantification of the mean fluorescence intensity of posteriorly localized Stau-GFP in *patronin*^{05252} and WT oocytes. ***$p = 0.0005$. Error bars indicate the SEM.

Scale bars represent 10 μm.

and these foci are the only visible source of cortical MTs, it seems most likely that the MTs are seeded from Patronin-stabilized MT minus-end stumps and not from de novo nucleation by the γ-tubulin ring complex.

Overexpression of the centriolar duplication factors dSAS6, dSas4, Sak/PLK4, and Ana2/STIL can promote the formation of acentriolar MTOCs in the oocyte (Dzhindzhev et al., 2010; Peel et al., 2007; Stevens et al., 2010). Moreover, expression of membrane-tethered Cep152/Asl and PLK4 is sufficient to induce formation of ectopic acentriolar MTOCs in mouse oocytes (Coelho et al., 2013). To test whether any of these acentriolar MTOC components are involved in the formation of the Shot/Patronin ncMTOCs, we co-expressed Cherry-Patronin with Asl-GFP (Figure 6B), Ana2-GFP (Figure 6C), dSas6-GFP, dSas4-GFP, and Sak-GFP (data not shown). None of these proteins co-localize with the Patronin foci, however, indicating that they are not components of the ncMTOCs (Figures 6B and 6C, and data not shown).

An alternative mechanism that can contribute to the formation of new MTs is the severing of existing MTs to generate minus ends that act as seeds for new microtubule growth (Baas and Ahmad, 1992; Lindeboom et al., 2013; Roll-Mecak and Vale, 2006). The mammalian Patronin orthologs, CAMSAP2 and CAMSAP3, associate with the microtubule severing protein, Katanin (Jiang et al., 2014). This association is conserved in *Drosophila*, as a protein trap insertion that labels endogenous Katanin 80 co-localizes with Patronin in the cortical foci in the oocyte and at the apical side of the follicle cells (Lowe et al.,

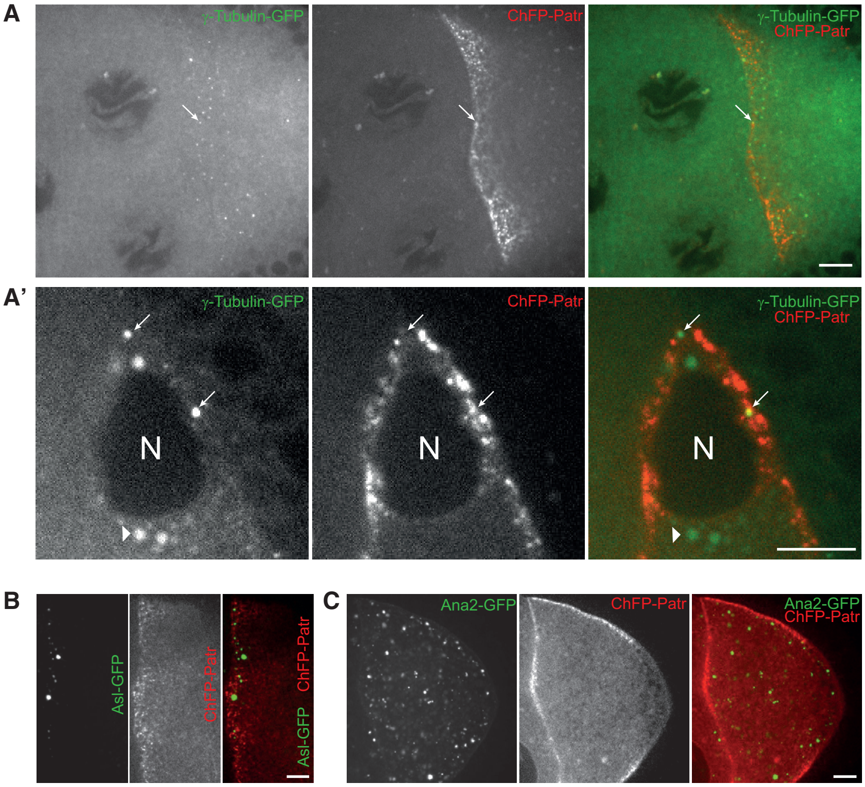

Figure 6. Patronin MTOCs and Centrosomal Components

(A and A′) Ectopically expressed γTub37C-GFP accumulates in cortical foci (A) and in the centrosomes around the nucleus (A′), but does not localize to the Patronin foci. Arrows point to γ-Tub-GFP-positive centrosomes. Arrowheads point to autofluorescent yolk particles. N, nucleus.

(B) Asl-GFP ectopically expressed under the control of *nanos*>Gal4 forms foci at oocyte cortex, but does not co-localize with Cherry-Patronin MTOCs.

(C) Ana2-GFP ectopically expressed under the control of *nanos*>Gal4 forms foci in the oocyte cytoplasm, but does not co-localize with the Cherry-Patronin MTOCs.

Scale bars represent 10 μm.

2014) (Figure S2). Furthermore, Katanin 80-YFP co-immunoprecipitates with Patronin from ovary extracts, confirming that it is a component of the cortical Patronin complex (Figure 3F). Thus, MT severing by Katanin may contribute to the generation of new MTs in the Patronin ncMTOCs.

Shot and Patronin Play a Role in the Formation of Apical-Basal MT Arrays in Follicle Epithelial Cells

In epithelial cells, noncentrosomal MTs form apical-basal arrays with their MT minus ends concentrated at the apical cortex (Bacallao et al., 1989; Jankovics and Brunner, 2006). The mammalian Patronin homolog, CAMSAP3, localizes to the apical cortex of mouse intestinal cells and human Caco2 cells, and mutation of *camsap3* leads to a random orientation of MTs (Toya et al., 2016). To test whether Patronin ncMTOCs play a similar role in the formation of the apical-basal array of MTs in *Drosophila* epithelia, we analyzed the localization of Patronin in the follicle cells, larval salivary glands, and male ejaculatory duct (Figures 3B, 7A, and S3). Patronin localizes apically in all three epithelia, forming multiple apical foci in the follicle cells, but is excluded from the adherens junctions (Figure 7B). Live imaging of EB1-GFP and Jupiter-GFP reveals that most MTs grow from the region of apical Patronin foci (Figure 7C and Movie S9). Although *capsap3* null cells contain relatively normal numbers of MTs, *patronin*^{05252} mutant cells have very few MTs (Figure 7D), presumably because it is the only copy of this gene in *Drosophila*. In addition, larger *patronin*^{05252} mutant clones often lead to tissue disorganization and multi-layering (Figures 7G and S4A). This suggests that Patronin apical foci act as ncMTOCs in epithelial cells and that they are crucial for tissue integrity.

Shot also localizes apically in the follicle cells and the embryonic salivary gland epithelium, and has been proposed to link acentrosomal MT minus ends to medial actomyosin, although this does not appear to require its ABD (Booth et al., 2014; Roper and Brown, 2003). This suggests that Shot may have similar role as an anchor of Patronin ncMTOCs in epithelial cells. In agreement with previous studies, we observed that Shot is strongly enriched at the apical side of the follicle cells, where it co-localizes with Patronin (Figures 2A and 7A). In homozygous clones of the ABD mutant, *shot*^{2A2}, Shot protein at the apical cortex is slightly reduced and the protein is found throughout the cytoplasm, indicating that the ABD contributes to efficient apical recruitment (Figure 7E).

To examine the role of Shot in MT organization, we generated clones of *shot*^{3}, a null mutation (Lee et al., 2000; Roper and Brown, 2003). Mutant clones lose the pronounced apical enrichment of MTs seen in wild-type cells and have fewer MTs than normal, with the remaining MTs mainly along the lateral cortex (Figures 7D and S4B). *shot*^{3} mutant cells contain more MTs than *patronin* mutant cells, however, and the absence of Shot does not disrupt the apical localization of Patronin (Figure 7F).

It has previously been shown that Patronin functions during spindle elongation in the embryo and in interphase S2 cells to protect MT minus ends from the depolymerizing kinesin, Klp10A (kinesin-13), as simultaneous knockdown of Klp10A and Patronin rescues the MT phenotype of Patronin knockdown alone (Goodwin and Vale, 2010; Wang et al., 2013). To ask whether Patronin also antagonizes KLP10a in epithelial cells, we examined the MT phenotype of *klp10a patronin* double-mutant clones. Loss of KLP10a partially rescues MT abundance in *patronin* mutant cells, but does not rescue the apical enrichment of MTs, resulting in an MT phenotype that is similar to that seen in *shot*^{3} (Figure 7G). By contrast, *klp10a* has no effect on MT density or organization in *shot*^{3} cells (Figure 7H). Thus, Patronin is required both to position MT minus ends apically and to protect them from depolymerization by Klp10A. Shot is not required for Patronin's activity in protecting MT minus ends, but the fact that *shot* and *klp10a patronin* mutants produce

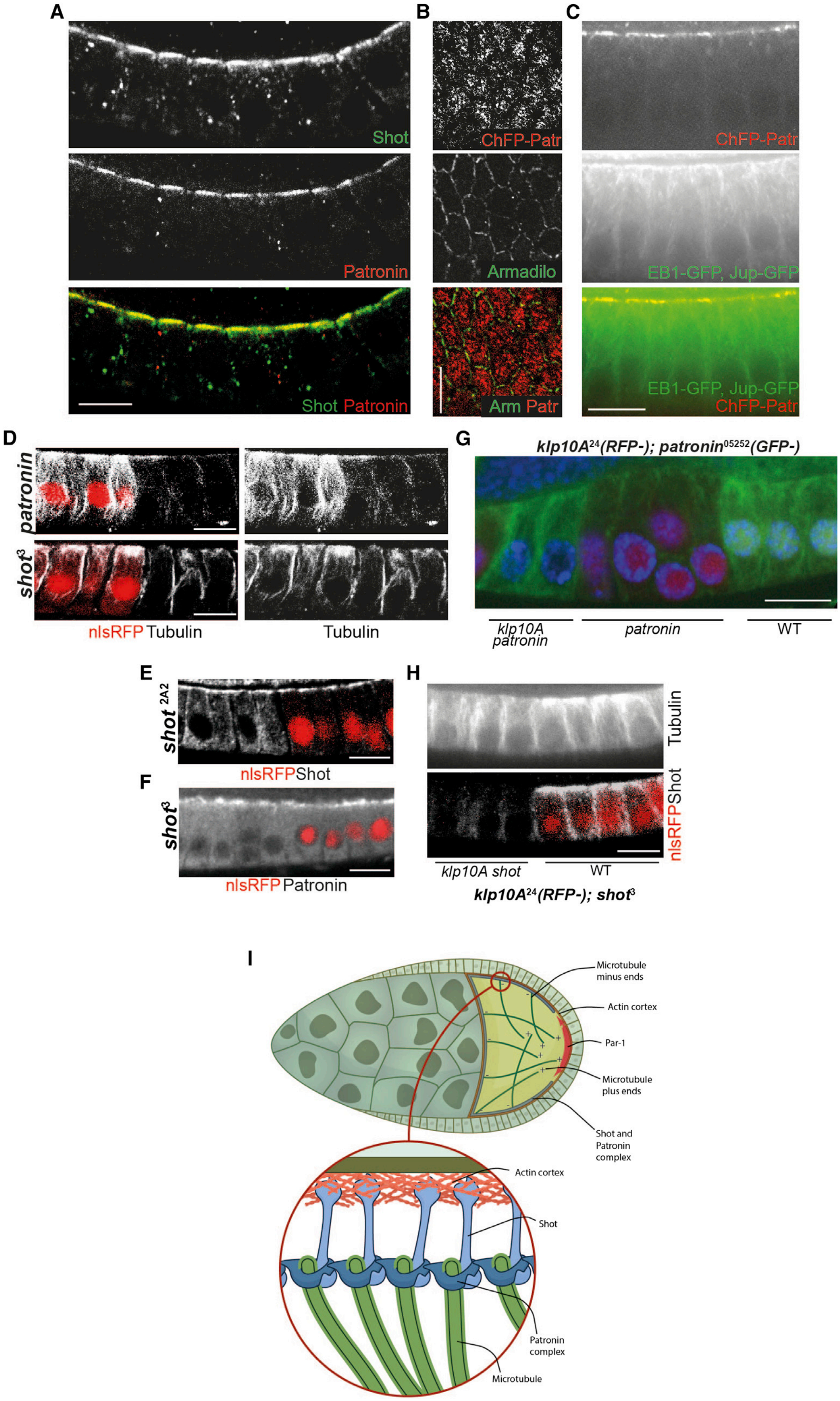

(legend on next page)

very similar defects in MT organization suggests that Shot and Patronin act in the same pathway to anchor MTs apically. We also tested whether Patronin functions in the oocyte to protect MT minus ends from depolymerization by Klp10A. However, *klp10a patronin* double-mutant germline clones show the same reduction in MT density as the *patronin* single mutant, suggesting that Klp10A plays little role in the germline (Figure S5).

DISCUSSION

The polarized arrangement of the MTs in the *Drosophila* oocyte depends on the posterior crescent of the Par-1 kinase, which excludes MT minus ends from the posterior cortex (Doerflinger et al., 2010; Parton et al., 2011). Here we show that Par-1 acts by preventing the association of Shot with the posterior actin cortex, thereby restricting the formation of noncentrosomal MTOCs to the anterior and lateral cortex. Computer modeling has shown that this asymmetric localization of MT minus ends is sufficient to explain the formation of the weakly polarized MT network that directs the transport of *oskar* mRNA to the posterior pole (Khuc Trong et al., 2015). Thus, the regulation of the interaction of Shot with the cortex by Par-1 transmits cortical PAR polarity into the polarization of the MT cytoskeleton that localizes the axis determinants (Figure 7I).

The mechanism by which Par-1 excludes Shot is unknown. The interaction of Shot with the cortex depends on its N-terminal calponin homology domains, which bind to F-actin (Lee and Kolodziej, 2002; Leung et al., 1999). Thus, Par-1 could phosphorylate Shot to inhibit its binding to the cortex. If this is the case, Par-1 would have to modify the activity or accessibility of the N-terminal ABD of Shot, as this domain recapitulates the posterior exclusion and cortical recruitment of the full-length protein. We have not detected any phosphorylation of the ABD by Par-1 in vitro, however, and it seems more likely that Par-1 acts by modifying the cortex to block the binding of Shot.

Shot and its vertebrate ortholog, MACF1, have previously been shown to interact with the MT plus-end tracking protein EB1 through their C-terminal SxIP motifs and with the MT lattice through their Gas2 and C-terminal domains (Alves-Silva et al., 2012; Applewhite et al., 2010; Honnappa et al., 2009; Kodama et al., 2003; Sun et al., 2001). Our results indicate that in addition to binding to MT plus ends and to the MT lattice, Shot also interacts with MT minus ends through its association with the Patronin/Katanin complex. The exact nature of the interaction between Shot and the Patronin complex is unclear, but Shot was found to interact with Katanin 60 in a high-throughput yeast two-hybrid screen (Giot et al., 2003). Thus, one possibility is that Katanin acts as a link between Shot and Patronin. Since Shot is exclusively cortical in the oocyte, the protein does not appear to bind to MT plus ends or along the body of MTs in this system. It will therefore be interesting to investigate whether the different modes of MT binding by Shot are mutually exclusive and how this is regulated.

Several models have been proposed to explain the formation of noncentrosomal MTs. Upon centrosome inactivation in post-mitotic *Drosophila* tracheal cells and *C. elegans* intestinal cells, γ-TuRC complexes and other pericentriolar material (PCM) components are released from the centrosome and transported toward the apical membrane, where they nucleate MT (Brodu et al., 2010; Feldman and Priess, 2012). Whole MTs released from the centrosome can also be delivered and anchored to the apical domain or cell junctions by Ninein (Lechler and Fuchs, 2007; Mogensen et al., 2000). Alternatively, new MTs can grow from MT ends generated by severing enzymes, a mechanism that is thought to be important in plant cells and neurons (Baas and Ahmad, 1992; Lindeboom et al., 2013; Roll-Mecak and Vale, 2006). Here, we present evidence that this latter mechanism is responsible for the formation of the MT array that directs *Drosophila* axis formation. Firstly, Shot/Patronin ncMTOCs contain stable minus ends even after treatment with the MT-depolymerizing drug, colcemid, as shown by the persistent recruitment of Tau-GFP and EB1-GFP to these foci. This is consistent with the ability of Patronin and CAMPSAPs to capture and stabilize minus ends of single MTs in vitro and in cells (Goodwin and Vale, 2010; Hendershott and Vale, 2014; Jiang et al., 2014; Meng et al., 2008). Secondly, MTs start to grow out in all directions from the Shot/Patronin foci immediately after colcemid inactivation. Indeed all visible growing MTs emanate from Patronin foci,

Figure 7. Shot and Patronin Are Required for MT Organization in the Epithelial Follicle Cells

(A) Shot and Patronin co-localize at the apical cortex of the follicle cells. An optical section through the epithelia monolayer with apical at the top and basal at the bottom. See also Figure S3.

(B) Follicle cells contain multiple apical Patronin foci. Top: view of the apical region of follicle cells expressing ubi>Cherry-Patronin. Patronin does not localize to the adherens junctions marked by Armadillo (green) staining.

(C) Apical Patronin foci co-localize with MTs. MTs were marked by ubi>EB1-GFP and Jupiter-GFP. The image is a temporal merge of several frames from Movie S9.

(D) MT organization in $patronin^{05252}$ and $shot^{3}$ mutant follicle cell clones marked by the loss of nuclear RFP (red). Top: $patronin^{05252}$ mutant cells contain many fewer microtubules than their heterozygous neighbors. Bottom: *shot* null mutant cells lose the apical enrichment of MTs, but retain lateral MTs. See also Figure S4.

(E) Shot protein is still enriched apically in $shot^{2A2}$ mutant follicle cells, but the protein is also diffusely distributed throughout the cytoplasm. $shot^{2A2}$ mutant cells were marked by the absence of nlsRFP.

(F) Shot is not required for the apical recruitment of Patronin in the follicle cells. $shot^{3}$ mutant cells were marked by the absence of nlsRFP.

(G) Patronin protects microtubule minus ends from the depolymerizing kinesin KLP10A in the follicle cells. The removal of KLP10A from $patronin^{05252}$ mutant cells partially rescues the loss of MTs caused by the *patronin* mutant alone. Mutant cells were marked by the absence of nlsGFP (*patronin*) and nlsRFP (*klp10A*). Double-mutant cells lack both GFP and RFP.

(H) Mutation of *klp10A* does not rescue the MT phenotype of $shot^{3}$ mutant clones. Double-mutant cells were marked by the absence of nlsRFP (*klp10A*) and by the loss of Shot staining (bottom).

(I) A model showing how Shot exclusion by Par-1 generates the polarized MT cytoskeleton in the oocyte. Par-1 is localized to the posterior of the oocyte, where it inhibits the association of Shot with the actin-rich cortex. Shot recruits Patronin to the anterior and lateral cortex to stabilize free MT minus ends and induce the formation of ncMTOCs that are the source of the MTs that localize *oskar* mRNA.

Scale bars represent 10 μm.

indicating that they are the principal source of MTs in the oocyte. Thirdly, the foci contain no detectable γ-tubulin and do not co-localize with PCM proteins. This is consistent with observations in Caco-2 cells, which showed that CAMSAP2 and CAMSAP3 do not co-localize with γ-tubulin and in the *C. elegans* epidermis, where PTRN-1 and γ-tubulin function in parallel pathways to assemble circumferential MTs (Tanaka et al., 2012; Wang et al., 2015).

Taken together, these results suggest a model in which the Shot/Patronin foci act as ncMTOCs by capturing and stabilizing MT minus-end stumps that then act as templates for new MT growth. One attractive feature of this model is that it uncouples MT organization from MT nucleation in both space and time. The Shot/Patronin complex bypasses the need to continually nucleate new MTs by preventing existing microtubules from completely depolymerizing. Thus, once a cell has nucleated sufficient MTs, it can maintain and reorganize its MT cytoskeleton by stabilizing MT minus-end stumps in appropriate locations and using these, rather than the γ-tubulin ring complex, to provide the seeds from which new MTs grow. The number of MTs can even increase in the absence of new MT nucleation if MT-severing proteins chop up existing MTs to produce new minus ends that can then be captured and stabilized. The presence of the severing protein, Katanin, in the Shot/Patronin foci is intriguing in this context, as it raises the possibility that it severs existing MTs to provide a local source of minus ends for Patronin to capture.

Shot and Patronin also co-localize at the apical cortex of the epithelial follicle cells, where they are required for apical-basal MT organization. This consistent with the recent observation that CAMSAP3 is required for the recruitment of MT minus ends to the apical cortex of mammalian intestinal epithelial cells (Toya et al., 2016). Thus, this function of Patronin has been evolutionarily conserved. Furthermore, the similarities between roles of Shot and Patronin in the oocyte and the follicle cells suggest that the complex may provide a general mechanism for organizing non-centrosomal MTs. The relationship between Shot and Patronin is different in the follicle cells compared with the oocyte, however, as Shot is not required for the apical recruitment of Patronin. Nevertheless, loss of either protein produces a very similar loss of apical MT and a reduction in overall MT density. Although we cannot rule out the possibility that they act in parallel pathways, this observation suggests that they collaborate to anchor MTs to the apical cortex. The combination of Patronin binding to the MT minus ends and Shot binding to the MT lattice may therefore provide a robust anchor to retain MTs at the apical cortex.

EXPERIMENTAL PROCEDURES

Colcemid Treatment

The protocol was modified from Parton et al. (2011). Flies were starved for 3 hr and then fed colcemid (Sigma) in yeast paste (66 μg/ml) for 2–3 hr. Ovaries were dissected and imaged as described below. Colcemid was inactivated with a brief UV pulse (3–5 s).

Imaging

For live imaging, ovaries were dissected and imaged in Voltalef oil 10S (VWR International) on an Olympus IX81 inverted microscope with a Yokogawa CSU22 spinning disk confocal imaging system (40× 1.35 NA Oil UPlanSApo, 60× 1.35 NA Oil UPlanSApo, and 100× 1.3 NA Oil UPlanSApo). Fixed preparations were imaged using Olympus IX81 (40× 1.35 NA Oil UPlanSApo, 60× 1.35 NA Oil UPlanSApo) and Zeiss LSM510 (40× NA 1.3 Oil Plan-NeoFluor) confocal microscopes. Images were collected with Olympus Fluoview, LSM510 AIM software, or MetaMorph software and processed using ImageJ. The oocyte cortex was imaged by collecting 10–15 z sections spaced 0.5 μm apart and then merging them.

Immunohistochemistry

Ovaries were fixed for 10 min in 10% paraformaldehyde and 2% Tween in PBS. Ovaries were then blocked with 10% BSA in PBS for 1 hr at room temperature. Ovaries were incubated with the primary antibody for 16 hr in PBS with 0.2% Tween and for 4 hr with the secondary antibody. In situ hybridizations were performed as previously described (Doerflinger et al., 2010). We used the following primary antibodies: mouse anti-α-tubulin fluorescein isothiocyanate at 1:250 (Sigma); mouse anti-Dynein heavy chain at 1:50 (DSHB); rabbit anti-Glued antibody raised against amino acid residues 1–400 of Glued and used at 1:100; mouse anti-DIG Cy3 at 1:200 (Jackson Immunoresearch), rabbit anti-Patronin (Goodwin and Vale, 2010) at 1:300 (gift from R. Vale, HHMI and UCSF, USA); mouse anti-Armadillo at 1:100 (DSHB); and guinea pig anti-Shot antibody raised against amino acid residues 2,602–3,640 (isoform PE) and used at 1:500. Conjugated secondary antibodies (Jackson Immunoresearch) were used at 1:100.

Molecular Biology

To generate a rescuing genomic *shot* transgene with C-terminal YFP tag, we used the PACMAN CH321-44M3 BAC clone (Venken et al., 2009) covering the entire *shot* locus. The BAC was modified using the *galK* positive/counter-selection cassette and recombineering (Warming et al., 2005). Transgenic flies were created by Genetivision.

The Patronin C-terminal YFP knockin was made by injecting nos>Cas9 embryos (Port et al., 2014) with a single guide RNA targeting the region of the stop codon in *patronin* (5′-GGCGCTTGTAATC**TAA**GCGG-3′, the stop codon is in bold) and a donor plasmid with 4-kb homology arms surrounding the Venus sequence.

pUASP-mKate-ABD was constructed by amplifying Shot ABD and mKate2 with the following primers: 5′-ATGTAGCGGCCGCCCGCGATGCCATTCAGAAGA-3′ and 5′-ATGTATCTAGATCAAATGTACGTGATGAGGGACT-3′; 5′ACGTGGTACCATGGTGAGCGAGCTGATT-3′ and 5′ATGTAGCGGCCGCGGAAGAGGAAGATCTGTGCCCCAGTTTGCT-3′. The amplified fragments were cloned into the pUASP vector (Rørth, 1998). The mutated Shot ABD was amplified with 5′-GATCAAACTGGACAACATACG-3′ and 5′-CGTATGTTGTCCAGTTTGATC-3′. Shot RE cDNA was obtained from A. Prokop (University of Manchester, UK).

For generation of pUASP-mCherry-Patronin and pUMAT-mCherry-Patronin, *patronin* RI and mCherry were amplified with 5′-ATGTAGGTACCATGGTGAGCAAGGGCGAGGAGGATAACA-3′ and 5′-GCATTCTAGATTAGATTACAAGCGCCATGTCTTTT-3′ from the pMT-mCherry-Patronin plasmid (Goodwin and Vale, 2010) (Addgene) and cloned into the pUASP vector (Rørth, 1998) and the pUMAT vector (Irion et al., 2006).

For generation of pUMAT-YFP-Patronin, *patronin* RI and YFP were amplified with 5′-ATGGACGAGCTGTACAAGCACCGGTATACAAGT-3′ and 5′-GCATTCTAGATTAGATTACAAGCGCCATGTCTTTT-3′, and 5′-TAGTAGGTACCCATGAGCAAGGGCGAGG-3′ and 5′-ACTTGTATACCGGTGCTTGTACAGCTCGTCCAT-3′, respectively and cloned into the pUMAT vector (Irion et al., 2006).

shot^{2A2} genomic DNA was isolated from homozygous embryos and larvae using the Gentra Puregene Cell Kit (Qiagen), and exonic regions were amplified by PCR and sequenced. Primer sequences are available on request.

SUPPLEMENTAL INFORMATION

Supplemental Information includes Supplemental Experimental Procedures, five figures, and nine movies and can be found with this article online at http://dx.doi.org/10.1016/j.devcel.2016.06.010.

AUTHOR CONTRIBUTIONS

D.N. performed most of the experiments and data analysis. A.R.F. performed immunoprecipitations. D.N. and D.St J. planned the experiments. D.N. and D.St J. conceived the project and wrote the manuscript.

ACKNOWLEDGMENTS

We are grateful to R. Vale, A. Prokop, N. Brown, J. Raff, S. Endow, K. McKim, the Kyoto Stock Center and the Bloomington Stock Center for flies and reagents. We thank N. Dawney for technical assistance and C. Flandoli for technical art work. This work was supported by a Wellcome Trust PRF to D.S.J. (080007) and by core support from the Wellcome Trust (092096) and Cancer Research UK (A14492). D.N. was supported by a postdoctoral fellowship from the Swedish Research Council. A.R.F. is funded by a University of Cambridge PhD studentship.

Received: February 2, 2016
Revised: May 3, 2016
Accepted: June 8, 2016
Published: July 11, 2016

REFERENCES

Akhmanova, A., and Steinmetz, M.O. (2015). Control of microtubule organization and dynamics: two ends in the limelight. Nat. Rev. Mol. Cell Biol. *16*, 711–726.

Alves-Silva, J., Sánchez-Soriano, N., Beaven, R., Klein, M., Parkin, J., Millard, T.H., Bellen, H.J., Venken, K.J.T., Ballestrem, C., Kammerer, R.A., et al. (2012). Spectraplakins promote microtubule-mediated axonal growth by functioning as structural microtubule-associated proteins and EB1-dependent +TIPs (tip interacting proteins). J. Neurosci. *32*, 9143–9158.

Applewhite, D.A., Grode, K.D., Keller, D., Zadeh, A., Slep, K.C., and Rogers, S.L. (2010). The spectraplakin Short stop is an actin-microtubule cross-linker that contributes to organization of the microtubule network. Mol. Biol. Cell *21*, 1714–1724.

Baas, P.W., and Ahmad, F.J. (1992). The plus ends of stable microtubules are the exclusive nucleating structures for microtubules in the axon. J. Cell Biol. *116*, 1231–1241.

Bacallao, R., Antony, C., Dotti, C., Karsenti, E., Stelzer, E.H., and Simons, K. (1989). The subcellular organization of Madin-Darby canine kidney cells during the formation of a polarized epithelium. J. Cell Biol. *109*, 2817–2832.

Baines, A.J., Bignone, P.A., King, M.D.A., Maggs, A.M., Bennett, P.M., Pinder, J.C., and Phillips, G.W. (2009). The CKK Domain (DUF1781) binds microtubules and defines the CAMSAP/ssp4 family of animal proteins. Mol. Biol. Evol. *26*, 2005–2014.

Bartolini, F., and Gundersen, G.G. (2006). Generation of noncentrosomal microtubule arrays. J. Cell Sci. *119*, 4155–4163.

Basto, R., Lau, J., Vinogradova, T., Gardiol, A., Woods, C.G., Khodjakov, A., and Raff, J.W. (2006). Flies without centrioles. Cell *125*, 1375–1386.

Bellen, H.J., Levis, R.W., Liao, G., He, Y., Carlson, J.W., Tsang, G., Evans-Holm, M., Hiesinger, P.R., Schulze, K.L., Rubin, G.M., et al. (2004). The BDGP gene disruption project: single transposon insertions associated with 40% of *Drosophila* genes. Genetics *167*, 761–781.

Booth, A.J.R., Blanchard, G.B., Adams, R.J., and Roper, K. (2014). A dynamic microtubule cytoskeleton directs medial actomyosin function during tube formation. Dev. Cell *29*, 562–576.

Brendza, R.P., Serbus, L.R., Saxton, W.M., and Duffy, J.B. (2002). Posterior localization of dynein and dorsal-ventral axis formation depend on kinesin in *Drosophila* oocytes. Curr. Biol. *12*, 1541–1545.

Brodu, V., Baffet, A.D., Le Droguen, P.-M., Casanova, J., and Guichet, A. (2010). A developmentally regulated two-step process generates a noncentrosomal microtubule network in *Drosophila* tracheal cells. Dev. Cell *18*, 790–801.

Chang, C.-W., Nashchekin, D., Wheatley, L., Irion, U., Dahlgaard, K., Montague, T.G., Hall, J., and St Johnston, D. (2011). Anterior-posterior axis specification in *Drosophila* oocytes:identification of novel *bicoid* and *oskar* mRNA localization factors. Genetics *188*, 883–896.

Chuang, M., Goncharov, A., Wang, S., Oegema, K., Jin, Y., and Chisholm, A.D. (2014). The microtubule minus-end-binding protein Patronin/PTRN-1 is required for axon regeneration in *C. elegans*. Cell Rep. *9*, 874–883.

Clark, I., Giniger, E., Ruohola-Baker, H., Jan, L.Y., and Jan, Y.-N. (1994). Transient posterior localization of a kinesin fusion protein reflects anteroposterior polarity of the *Drosophila* oocyte. Curr. Biol. *4*, 289–300.

Clark, I.E., Jan, L.Y., and Jan, Y.N. (1997). Reciprocal localization of Nod and kinesin fusion proteins indicates microtubule polarity in the *Drosophila* oocyte, epithelium, neuron and muscle. Development *124*, 461–470.

Coelho, P.A., Bury, L., Sharif, B., Riparbelli, M.G., Fu, J., Callaini, G., Glover, D.M., and Zernicka-Goetz, M. (2013). Spindle formation in the mouse embryo requires Plk4 in the absence of centrioles. Dev. Cell *27*, 586–597.

Doerflinger, H., Vogt, N., Torres, I.L., Mirouse, V., Koch, I., Nüsslein-Volhard, C., and St Johnston, D. (2010). Bazooka is required for polarisation of the *Drosophila* anterior-posterior axis. Development *137*, 1765–1773.

Dzhindzhev, N.S., Yu, Q.D., Weiskopf, K., Tzolovsky, G., Cunha-Ferreira, I., Riparbelli, M., Rodrigues-Martins, A., Bettencourt-Dias, M., Callaini, G., and Glover, D.M. (2010). Asterless is a scaffold for the onset of centriole assembly. Nature *467*, 714–718.

Feldman, J.L., and Priess, J.R. (2012). A role for the centrosome and PAR-3 in the hand-off of MTOC function during epithelial polarization. Curr. Biol. *22*, 575–582.

Giot, L., Bader, J.S., Brouwer, C., Chaudhuri, A., Kuang, B., Li, Y., Hao, Y.L., Ooi, C.E., Godwin, B., Vitols, E., et al. (2003). A protein interaction map of *Drosophila* melanogaster. Science *302*, 1727–1736.

Goodwin, S.S., and Vale, R.D. (2010). Patronin regulates the microtubule network by protecting microtubule minus ends. Cell *143*, 263–274.

Hanein, D., Volkmann, N., Goldsmith, S., Michon, A.M., Lehman, W., Craig, R., DeRosier, D., Almo, S., and Matsudaira, P. (1998). An atomic model of fimbrin binding to F-actin and its implications for filament crosslinking and regulation. Nat. Struct. Biol. *5*, 787–792.

Hendershott, M.C., and Vale, R.D. (2014). Regulation of microtubule minus-end dynamics by CAMSAPs and Patronin. Proc. Natl. Acad. Sci. USA *111*, 5860–5865.

Honnappa, S., Gouveia, S.M., Weisbrich, A., Damberger, F.F., Bhavesh, N.S., Jawhari, H., Grigoriev, I., van Rijssel, F.J.A., Buey, R.M., Lawera, A., et al. (2009). An EB1-binding motif acts as a microtubule tip localization signal. Cell *138*, 366–376.

Irion, U., Adams, J., Chang, C.-W., and St Johnston, D. (2006). Miranda couples *oskar* mRNA/Staufen complexes to the *bicoid* mRNA localization pathway. Dev. Biol. *297*, 522–533.

Jankovics, F., and Brunner, D. (2006). Transiently reorganized microtubules are essential for zippering during dorsal closure in *Drosophila melanogaster*. Dev. Cell *11*, 375–385.

Januschke, J., Gervais, L., Gillet, L., Keryer, G., Bornens, M., and Guichet, A. (2006). The centrosome-nucleus complex and microtubule organization in the *Drosophila* oocyte. Development *133*, 129–139.

Jiang, K., Hua, S., Mohan, R., Grigoriev, I., Yau, K.W., Liu, Q., Katrukha, E.A., Altelaar, A.F.M., Heck, A.J.R., Hoogenraad, C.C., et al. (2014). Microtubule minus-end stabilization by polymerization-driven CAMSAP deposition. Dev. Cell *28*, 295–309.

Karpova, N., Bobinnec, Y., Fouix, S., Huitorel, P., and Debec, A. (2006). Jupiter, a new *Drosophila* protein associated with microtubules. Cell Motil. Cytoskeleton *63*, 301–312.

Khuc Trong, P., Doerflinger, H., Dunkel, J., St Johnston, D., and Goldstein, R.E. (2015). Cortical microtubule nucleation can organise the cytoskeleton of *Drosophila* oocytes to define the anteroposterior axis. Elife *4*, e06088.

Kodama, A., Karakesisoglou, I., Wong, E., and Vaezi, A. (2003). ACF7 an essential integrator of microtubule dynamics. Cell *115*, 343–354.

Lechler, T., and Fuchs, E. (2007). Desmoplakin: an unexpected regulator of microtubule organization in the epidermis. J. Cell Biol. *176*, 147–154.

Lee, S., and Kolodziej, P. (2002). Short Stop provides an essential link between F-actin and microtubules during axon extension. Development *129*, 1195–1204.

Lee, S., Harris, K.L., Whitington, P.M., and Kolodziej, P.A. (2000). *Short stop* is allelic to *kakapo*, and encodes rod-like cytoskeletal-associated proteins required for axon extension. J. Neurosci. *20*, 1096–1108.

Leung, C., Sun, D., Zheng, M., Knowles, D., and Liem, R. (1999). Microtubule actin cross-linking factor (MACF): a hybrid of Dystonin and Dystrophin that can interact with the actin and microtubule cytoskeletons. J. Cell Biol. *147*, 1275–1286.

Lindeboom, J.J., Nakamura, M., Hibbel, A., Shundyak, K., Gutierrez, R., Ketelaar, T., Emons, A.M.C., Mulder, B.M., Kirik, V., and Ehrhardt, D.W. (2013). A mechanism for reorientation of cortical microtubule arrays driven by microtubule severing. Science *342*, 1245533.

Lowe, N., Rees, J.S., Roote, J., Ryder, E., Armean, I.M., Johnson, G., Drummond, E., Spriggs, H., Drummond, J., Magbanua, J.P., et al. (2014). Analysis of the expression patterns, subcellular localisations and interaction partners of *Drosophila* proteins using a pigP protein trap library. Development *141*, 3994–4005.

Marcette, J.D., Chen, J.J., Nonet, M.L., and Hobert, O. (2014). The *Caenorhabditis elegans* microtubule minus-end binding homolog PTRN-1 stabilizes synapses and neurites. Elife *3*, e01637.

Meng, W., Mushika, Y., Ichii, T., and Takeichi, M. (2008). Anchorage of microtubule minus ends to adherens junctions regulates epithelial cell-cell contacts. Cell *135*, 948–959.

Mogensen, M.M., Malik, A., Piel, M., Bouckson-Castaing, V., and Bornens, M. (2000). Microtubule minus-end anchorage at centrosomal and non-centrosomal sites: the role of Ninein. J. Cell Sci. *113*, 3013–3023.

Nguyen, M.M., Stone, M.C., and Rolls, M.M. (2011). Microtubules are organized independently of the centrosome in *Drosophila* neurons. Neural Dev. *6*, 38.

Palacios, I.M., and St Johnston, D. (2002). Kinesin light chain-independent function of the Kinesin heavy chain in cytoplasmic streaming and posterior localisation in the *Drosophila* oocyte. Development *129*, 5473–5485.

Parton, R.M., Hamilton, R.S., Ball, G., Yang, L., Cullen, C.F., Lu, W., Ohkura, H., and Davis, I. (2011). A PAR-1-dependent orientation gradient of dynamic microtubules directs posterior cargo transport in the *Drosophila* oocyte. J. Cell Biol. *194*, 121–135.

Peel, N., Stevens, N.R., Basto, R., and Raff, J.W. (2007). Overexpressing centriole-replication proteins in vivo induces centriole overduplication and de novo formation. Curr. Biol. *17*, 834–843.

Port, F., Chen, H.-M., Lee, T., and Bullock, S.L. (2014). Optimized CRISPR/Cas tools for efficient germline and somatic genome engineering in *Drosophila*. Proc. Natl. Acad. Sci. USA *111*, E2967–E2976.

Richardson, C.E., Spilker, K.A., Cueva, J.G., Perrino, J., Goodman, M.B., Shen, K., and Hobert, O. (2014). PTRN-1, a microtubule minus end-binding CAMSAP homolog, promotes microtubule function in *Caenorhabditis elegans* neurons. Elife *3*, e01498.

Rogers, G.C., Rusan, N.M., Peifer, M., and Rogers, S.L. (2008). A multicomponent assembly pathway contributes to the formation of acentrosomal microtubule arrays in interphase *Drosophila* cells. Mol. Biol. Cell *19*, 3163–3178.

Roll-Mecak, A., and Vale, R.D. (2006). Making more microtubules by severing: a common theme of noncentrosomal microtubule arrays? J. Cell Biol. *175*, 849–851.

Roper, K., and Brown, N.H. (2003). Maintaining epithelial integrity: a function for gigantic spectraplakin isoforms in adherens junctions. J. Cell Biol. *162*, 1305–1315.

Roper, K., and Brown, N.H. (2004). A spectraplakin is enriched on the fusome and organizes microtubules during oocyte specification in *Drosophila*. Curr. Biol. *14*, 99–110.

Rørth, P. (1998). Gal4 in the *Drosophila* female germline. Mech. Dev. *78*, 113–118.

Shulman, J.M., Benton, R., and St Johnston, D. (2000). The *Drosophila* homolog of *C. elegans* PAR-1 organizes the oocyte cytoskeleton and directs oskar mRNA localization to the posterior pole. Cell *101*, 377–388.

St Johnston, D. (2005). Moving messages: the intracellular localization of mRNAs. Nat. Rev. Mol. Cell Biol. *6*, 363–375.

Stevens, N.R., Raposo, A.A.S.F., Basto, R., St Johnston, D., and Raff, J.W. (2007). From stem cell to embryo without centrioles. Curr. Biol. *17*, 1498–1503.

Stevens, N.R., Dobbelaere, J., Brunk, K., Franz, A., and Raff, J.W. (2010). *Drosophila* Ana2 is a conserved centriole duplication factor. J. Cell Biol. *188*, 313–323.

Stiess, M., Maghelli, N., Kapitein, L.C., Gomis-Rüth, S., Wilsch-Bräuninger, M., Hoogenraad, C.C., Tolić-Nørrelykke, I.M., and Bradke, F. (2010). Axon extension occurs independently of centrosomal microtubule nucleation. Science *327*, 704–707.

Sun, D., Leung, C.L., and Liem, R.K. (2001). Characterization of the microtubule binding domain of microtubule actin crosslinking factor (MACF): identification of a novel group of microtubule associated proteins. J. Cell Sci. *114*, 161–172.

Tanaka, N., Meng, W., Nagae, S., and Takeichi, M. (2012). Nezha/CAMSAP3 and CAMSAP2 cooperate in epithelial-specific organization of noncentrosomal microtubules. Proc. Natl. Acad. Sci. USA *109*, 20029–20034.

Theurkauf, W.E., Smiley, S., Wong, M.L., and Alberts, B.M. (1992). Reorganization of the cytoskeleton during *Drosophila* oogenesis: implications for axis specification and intercellular transport. Development *115*, 923–936.

Toya, M., Kobayashi, S., Kawasaki, M., Shioi, G., Kaneko, M., Ishiuchi, T., Misaki, K., Meng, W., and Takeichi, M. (2016). CAMSAP3 orients the apical-to-basal polarity of microtubule arrays in epithelial cells. Proc. Natl. Acad. Sci. USA *113*, 332–337.

Venken, K.J.T., Carlson, J.W., Schulze, K.L., Pan, H., He, Y., Spokony, R., Wan, K.H., Koriabine, M., de Jong, P.J., White, K.P., et al. (2009). Versatile P [acman] BAC libraries for transgenesis studies in *Drosophila melanogaster*. Nat. Methods *6*, 431–434.

Wang, H., Brust-Mascher, I., Civelekoglu-Scholey, G., and Scholey, J.M. (2013). Patronin mediates a switch from kinesin-13-dependent poleward flux to anaphase B spindle elongation. J. Cell Biol. *4*, 1343.

Wang, S., Wu, D., Quintin, S., Green, R.A., Cheerambathur, D.K., Ochoa, S.D., Desai, A., and Oegema, K. (2015). NOCA-1 functions with γ-tubulin and in parallel to Patronin to assemble non-centrosomal microtubule arrays in *C. elegans*. Elife *4*, e08649.

Warming, S., Costantino, N., Court, D.L., Jenkins, N.A., and Copeland, N.G. (2005). Simple and highly efficient BAC recombineering using galK selection. Nucl. Acids Res. *33*, e36.

Yau, K.W., van Beuningen, S.F.B., Cunha-Ferreira, I., Cloin, B.M.C., van Battum, E.Y., Will, L., Schätzle, P., Tas, R.P., van Krugten, J., Katrukha, E.A., et al. (2014). Microtubule minus-end binding protein CAMSAP2 controls axon specification and dendrite development. Neuron *82*, 1058–1073.

Zheng, J., Furness, D., Duan, C., Miller, K.K., Edge, R.M., Chen, J., Homma, K., Hackney, C.M., Dallos, P., and Cheatham, M.A. (2013). Marshalin, a microtubule minus-end binding protein, regulates cytoskeletal structure in the organ of Corti. Biol. Open *2*, 1192–1202.

Zimyanin, V.L., Belaya, K., Pecreaux, J., Gilchrist, M.J., Clark, A., Davis, I., and St Johnston, D. (2008). In vivo imaging of *oskar* mRNA transport reveals the mechanism of posterior localization. Cell *134*, 843–853.

Developmental Cell, Volume *38*

Supplemental Information

Patronin/Shot Cortical Foci Assemble the Noncentrosomal Microtubule Array that Specifies the *Drosophila* Anterior-Posterior Axis

Dmitry Nashchekin, Artur Ribeiro Fernandes, and Daniel St Johnston

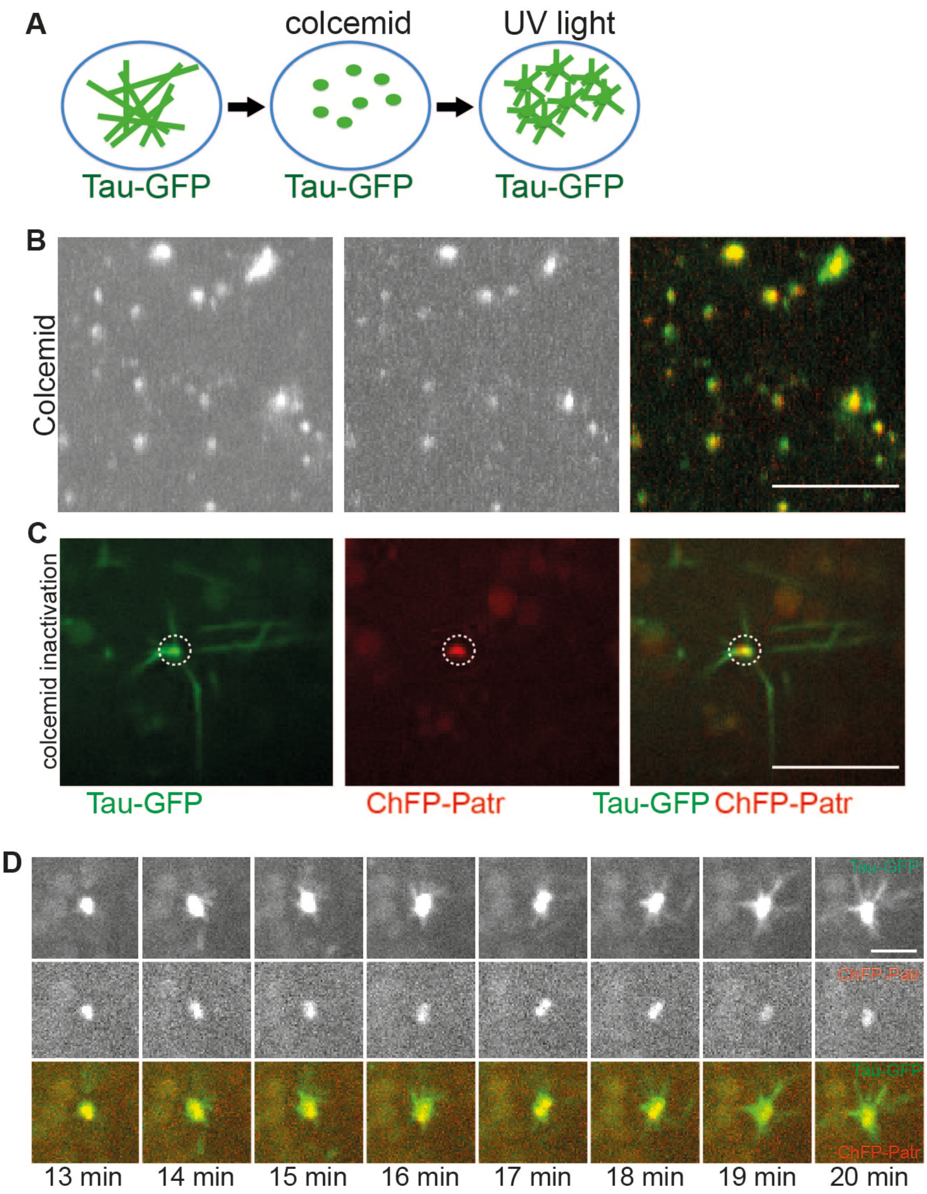

Figure S1. Patronin foci are cortical noncentrosomal MTOCs.
Related to Figure 4.
(A) Diagram of the microtubule re-growth assay.
(B-C) A close-up of a region of the lateral cortex of an oocyte expressing Tau-GFP from the maternal α4tubulin promoter and UAS-Cherry-Patronin driven by nanos>Gal4.
(B) Patronin foci co-localise with the microtubule-binding protein Tau-GFP in the presence of the microtubule-depolymerising drug, colcemid.
(C) After colcemid inactivation with UV light, Tau-GFP labelled microtubules re-grow from the Cherry-Patronin foci, indicating that the latter represent MTOCs.
(D) Still images from Movie S5 showing an example of an active Cherry-Patronin MTOC after colcemid inactivation. The time in minutes since colcemid inactivation is shown at the bottom. Scale bars represent 5 μm.

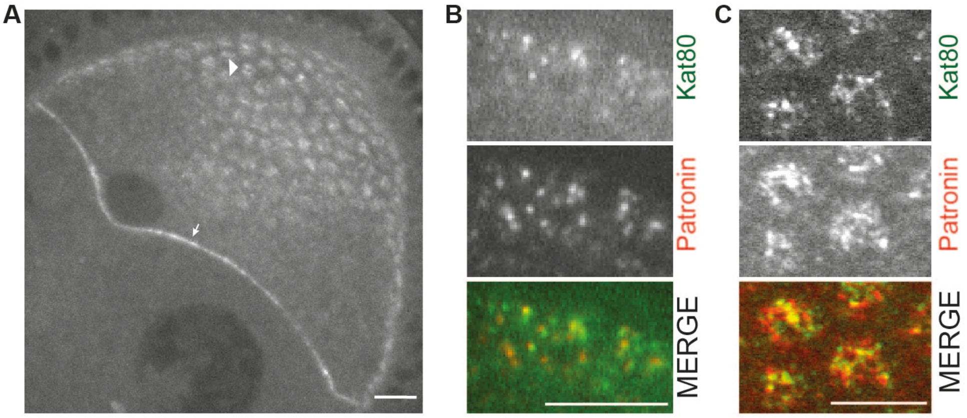

Figure S2. A protein trap insertion in Katanin 80 co-localises with Patronin ncMTOCs. Related to Figure 6.

(A) Katanin 80-YFP localises to the oocyte anterior and to the apical cortex of the follicle cells. The arrow points to the anterior and the arrowhead indicates the apical side of a follicle cell.

(B) Katanin 80 co-localises with Patronin in foci at the cortex of the oocyte.

(C) Katanin 80 and Patronin also co-localise at the apical side of the follicle cells.

Scale bars 10 μm.

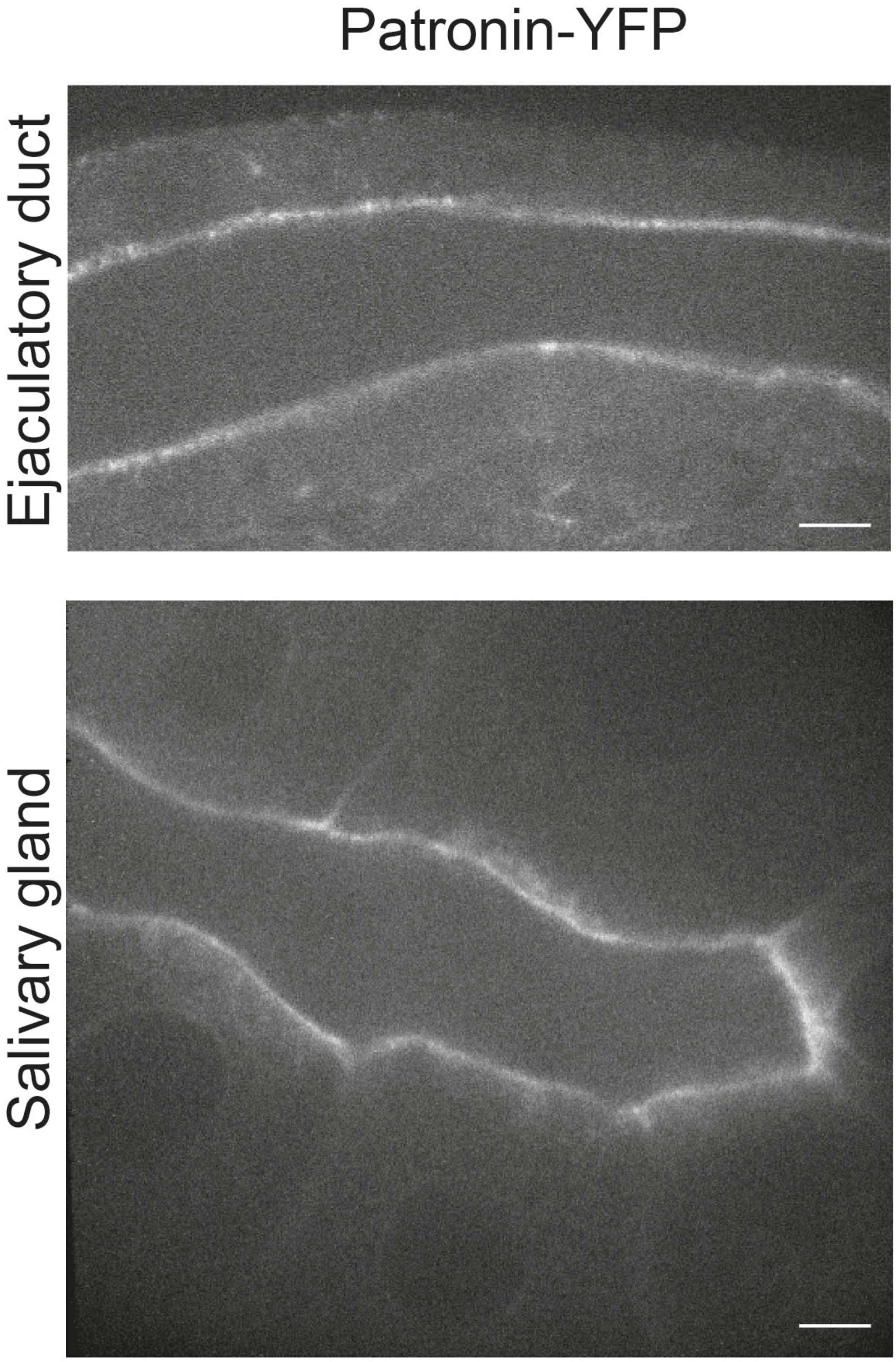

Figure S3. Patronin localises to the apical cortex of epithelial cells. Related to Figure 7.
Images of Patronin-YFP in the ejaculatory duct and in the larval salivary gland. Scale bars 10 μm.

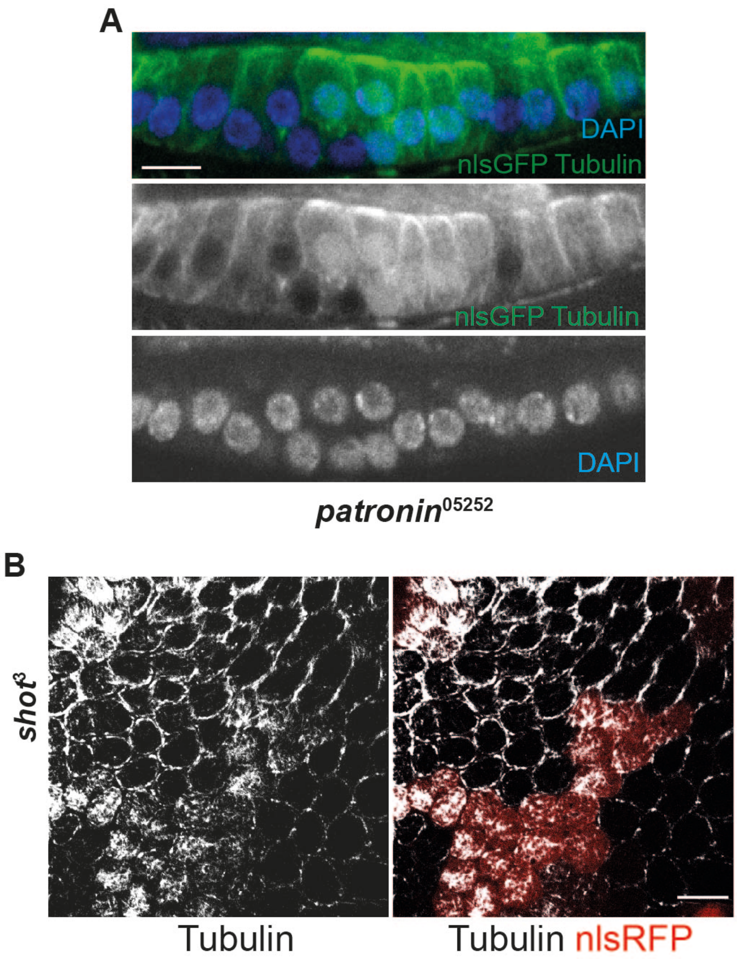

Figure S4. *patronin* and *shot* mutants disrupt the follicle cell epithelial monolayer. Related to Figure 7.
(A) *patronin*^{05252} mutant cells marked by the loss of nlsGFP lead to a disruption of epithelial organisation.
(B) A top view of the apical surface of the follicular epithelium containing a *shot*^{3} mutant clone marked by the loss of nlsRFP. The mutant cells lose the apical enrichment of microtubules that is visible in the adjacent wild-type cells.
Scale bars 10 μm.

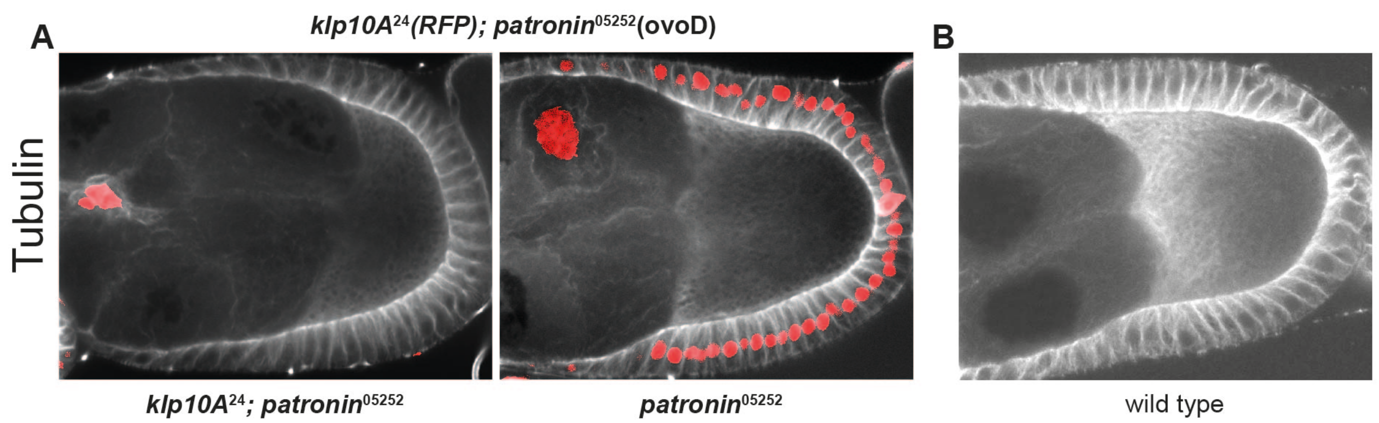

Figure S5. Removal of KLP10A does not rescue the *patronin*^{05252} microtubule phenotype in the oocyte. Related to Figure 7G.

(A) Germline clones mutant for both *klp10a* and *patronin*^{05252} (left) or *patronin*^{05252} only (right) stained with anti-tubulin. The *patronin*^{05252} clones were generated using the ovoD system, and *klp10a* germline clones were marked by the absence of nlsRFP in the nurse cells (left panel only; the red nuclei are heterozygous border cells).

(B) A wild type oocyte stained with anti-tubulin.

Supplemental Experimental Procedures

Fly stocks and genetics. *white*^{1118} was used as a wild-type stock throughout. The following mutant alleles and transgenic lines were used: *shot*^{2A2} (Chang et al., 2011), *shot*^{3} (Roper and Brown, 2003)(gift from N. Brown, University of Cambridge, UK), *patronin*^{EY05252} (Kyoto: 114436), *klp10A*^{24} (Radford et al., 2012) (gift from K. McKim, Waksman Institute and the State University of New Jersey, USA), *par-1*^{w3}/*par-1*^{6323} (Shulman et al., 2000), a protein trap line insertion in Jupiter-eGFP(Morin et al., 2001), maternal a4tubulin>GFP-Staufen(Martin et al., 2003), maternal a4tubulin>Tau-GFP(Doerflinger et al., 2003), UASp>GFP-EB1(Zhao et al., 2012), UASp>GFP-Par-1^{T786A} (Doerflinger et al., 2010), ncd>g-Tubulin37C-GFP (gift from S. Endow, Duke University, USA), UASp>Asl-GFP and UASp>Ana2-GFP(Stevens et al., 2010) (gift from J. Raff, University of Oxford, UK), ubi>EB1-GFP(Shimada et al., 2006), a protein trap insertion in Katanin80-YFP(Lowe et al., 2014). The mat-a4tub>Gal4-VP16 and nanos>Gal4 drivers were used to express UAS constructs in the germ line. Clones were generated with FRT G13, FRT G13 ovoD, FRT G13 RFP, FRT G13 GFP, FRT 19A RFP (Bloomington Stock Center) using the heat shock Flp/FRT system(Chou and Perrimon, 1992). The lethality and mutant phenotypes of *shot*^{2A2}/*shot*^{3} were rescued by a *shot* genomic BAC construct.

Co-immunoprecipitation. Ovarian extracts were prepared from wild-type, Shot-YFP and Kat80-YFP transgenic flies by dissecting ovaries (20-25 flies per genotype) into Schneider medium (Sigma) supplemented with 2.5% calf serum, followed by homogenization in ice cold PBS supplemented with 0.5% NP40, Complete Protease Inhibitor cocktail (Roche). The lipid-free fraction of the supernatant was collected after centrifugation at 16 000 g for 10 min at 4 ºC. Shot-YFP and Katanin 80-YFP were purified from 500 µL of ovarian extract using 5 µL of GFP-Trap®_MA (Chromotek) magnetic beads for 1 hr at 4 ºC with rotation. Input (4% of the extract) and bound fractions were analysed by SDS-PAGE and western blotting, using NuPage, 3-8% Tris-Acetate gels (ThermoFisher). Patronin was detected using 1:10000 rabbit anti-Patronin antibody(Goodwin and Vale, 2010) (gift from R. Vale, HHMI and UCSF, USA) and the ECL prime detection kit (GE LifeSciences).

SUPLEMENTAL REFERENCES

Chou, T.B., and Perrimon, N. (1992). Use of a yeast site-specific recombinase to produce female germline chimeras in Drosophila. Genetics *131*, 643–653.

Doerflinger, H., Benton, R., Shulman, J., and Johnston, D. (2003). The role of PAR-1 in regulating the polarised microtubule cytoskeleton in the Drosophila follicular epithelium. Development *130*, 3965.

Martin, S.G., Leclerc, V., Smith-Litière, K., and St Johnston, D. (2003). The identification of novel genes required for Drosophila anteroposterior axis formation in a germline clone screen using GFP-Staufen. Development *130*, 4201–4215.

Morin, X., Daneman, R., Zavortink, M., and Chia, W. (2001). A protein trap strategy to detect GFP-tagged proteins expressed from their endogenous loci in Drosophila. Proc Natl Acad Sci USA *98*, 15050–15055.

Radford, S.J., Harrison, A.M., and McKim, K.S. (2012). Microtubule-Depolymerizing Kinesin KLP10A Restricts the Length of the Acentrosomal Meiotic Spindle in Drosophila Females. Genetics *192*, 431–440.

Shimada, Y., Yonemura, S., Ohkura, H., Strutt, D., and Uemura, T. (2006). Polarized transport of Frizzled along the planar microtubule arrays in Drosophila wing

epithelium. Dev Cell *10*, 209–222.

Zhao, T., Graham, O.S., Raposo, A., and St Johnston, D. (2012). Growing microtubules push the oocyte nucleus to polarize the Drosophila dorsal-ventral axis. Science *336*, 999–1003.

SUPPLEMENTAL MOVIES

Movie S1 A time-lapse video of the microtubule-associated protein Jupiter-GFP in a wild-type stage 9 oocyte, showing the anterior-posterior gradient of microtubules. Related to Figure 1E.
Images were collected every 20 seconds on a spinning disc confocal microscope. The video is shown at 20 frames/sec.

Movie S2 A time-lapse video of Jupiter-GFP in a $shot^{2A2}$ mutant stage 9 oocyte. Related to Figure 1E.
The microtubules are not enriched anteriorly and distribute more evenly throughout the oocyte. Images were collected every 30 seconds. The video is shown at 13.3 frames/sec.

Movie S3 Cherry-Patronin localises to the anterior/lateral cortex of wild-type oocytes (left) and throughout the cytoplasm in $shot^{2A2}$ mutant oocytes (right). Related to Figure 3D. Images were collected every 10 seconds. The video is shown at 15 frames/s.

Movie S4 Microtubules grow from Cherry-Patronin foci after colcemid inactivation. Related to Figure 4C.
The growing plus ends of microtubules are labelled with EB1-GFP. The left panel shows EB1-GFP and the right panel Cherry-Patronin. The first 10 frames were taken before colcemid inactivation by a pulse of UV light. Images were collected every 3 seconds. The video is shown at 15 frames/s.

Movie S5 Microtubules grow from Cherry-Patronin foci after colcemid inactivation. Related to Figure 4C and Figure S1.
Microtubules are labelled with Tau-GFP. Images were collected every 1 minute. The video is shown at 10 frames/s.

Movie S6 Cherry-Patronin foci act as a source of growing MTs under steady state conditions in the absence of colcemid. Related to Figure 4D.
Microtubules are labelled with EB1-GFP. Cherry-Patronin is pseudocoloured in green. Images were collected every 1.6 seconds. The video is shown at 20 frames/s.

Movie S7 Mislocalisation of ncMTOCs labelled by EB1-GFP in $shot^{2A2}$. Related to Figure 4F.
Wild-type (left), *shot* (right). The first 10 frames were taken before colcemid inactivation by a pulse of UV light. Images were collected every second. The video is shown at 15 frames/s.

Movie S8 Microtubules labelled by EB1-GFP grow from the few ncMTOCs that form in $patronin^{05252}$ mutant oocytes. Related to Figure 5C.
Wild type (left), $patronin^{05252}$ (right). The first 4 frames were taken before colcemid inactivation by a pulse of UV light. Images were collected every second. The video is shown at 15 frames/s.

Movie S9 Microtubules grow from the apical cortex of the follicle cells where Cherry-Patronin foci (red) are localised. Related to Figure 7C.
Microtubules are labelled with EB1-GFP and Jupiter-GFP. Images were collected every 1.3 seconds. The video is shown at 30 frames/s.
